# Factors associated with successful dietary changes in an energy-reduced Mediterranean diet intervention: a longitudinal analysis in the PREDIMED-Plus trial

**DOI:** 10.1007/s00394-021-02697-8

**Published:** 2021-11-30

**Authors:** Cesar I. Fernandez-Lazaro, Estefanía Toledo, Pilar Buil-Cosiales, Jordi Salas-Salvadó, Dolores Corella, Montserrat Fitó, J. Alfredo Martínez, Ángel M. Alonso-Gómez, Julia Wärnberg, Jesús Vioque, Dora Romaguera, José López-Miranda, Ramon Estruch, Francisco J. Tinahones, José Lapetra, Luís Serra-Majem, Aurora Bueno-Cavanillas, Josep A. Tur, Vicente Martín Sánchez, Xavier Pintó, Miguel Delgado-Rodríguez, Pilar Matía-Martín, Josep Vidal, Emilio Ros, Clotilde Vázquez, Lidia Daimiel, Beatriz SanJulián, Jesús F. García-Gavilán, Jose V. Sorlí, Olga Castañer, M. Ángeles Zulet, Lucas Tojal-Sierra, Napoleón Pérez-Farinós, Alejandro Oncina-Canovas, Manuel Moñino, Antonio Garcia-Rios, Emilio Sacanella, Rosa M. Bernal-Lopez, José Manuel Santos-Lozano, Zenaida Vázquez-Ruiz, Jananee Muralidharan, Carolina Ortega-Azorín, Alberto Goday, Cristina Razquin, Leire Goicolea-Güemez, Miguel Ruiz-Canela, Nerea Becerra-Tomás, Helmut Schröder, Miguel A. Martínez González, for the PREDIMED-Plus investigators

**Affiliations:** 1 Department of Preventive Medicine and Public Health, NavarraUniversity of Navarra, IdiSNA, C/ Irunlarrea, 31008 Pamplona, Spain; 2 Centro de Investigación Biomédica en Red Fisiopatología de la Obesidad y la Nutrición (CIBEROBN), Institute of Health Carlos III, Madrid, Spain; 3 Servicios de Atención Primaria, Navarra Regional Health Service (Osasunbidea), IdiSNA, Pamplona, Spain; 4 Universitat Rovira I Virgili, Departament de Bioquímica i Biotecnologia, Unitat de Nutrició Humana, Reus, Spain; 5 Nutrition Unit, University Hospital of Sant Joan de Reus, Reus, Spain; 6 Institut d’Investigació Sanitària Pere Virgili (IISPV), Reus, Spain; 7 Department of Preventive Medicine, University of Valencia, Valencia, Spain; 8 Unit of Cardiovascular Risk and Nutrition, Institut Hospital del Mar de Investigaciones Médicas Municipal d`Investigació Médica (IMIM), Barcelona, Spain; 9 Department of Nutrition, Food Sciences, and Physiology, Center for Nutrition Research, University of Navarra, Pamplona, Spain; 10 Cardiometabolic Nutrition Group, IMDEA Food, CEI UAM + CSIC, Madrid, Spain; 11 Bioaraba Health Research Institute, Cardiovascular, Respiratory and Metabolic Area, Osakidetza Basque Health Service, Araba University Hospital, University of the Basque Country UPV/EHU, Vitoria-Gasteiz, Spain; 12 Epi-Phaan Research Group, School of Health Sciences, Universidad de Málaga, Instituto de Investigación Biomédica de Málaga (IBIMA), 29071 Málaga, Spain; 13 CIBER de Epidemiología y Salud Pública (CIBERESP), Instituto de Salud Carlos III, Madrid, Spain; 14 Instituto de Investigación Sanitaria y Biomédica de Alicante (ISABIAL-UMH), Alicante, Spain; 15 Health Research Institute of the Balearic Islands (IdISBa), Palma de Mallorca, Spain; 16 Department of Internal Medicine, Maimonides Biomedical Research Institute of Cordoba (IMIBIC), Reina Sofia University Hospital, University of Cordoba, Cordoba, Spain; 17 Department of Internal Medicine, Institut d’Investigacions Biomèdiques August Pi Sunyer (IDIBAPS), Hospital Clinic, University of Barcelona, Barcelona, Spain; 18 Department of Endocrinology, Virgen de la Victoria Hospital, Instituto de Investigación Biomédica de Málaga (IBIMA), University of Málaga, Málaga, Spain; 19 Department of Family Medicine, Research Unit, Distrito Sanitario Atención Primaria Sevilla, Sevilla, Spain; 20 Research Institute of Biomedical and Health Sciences (IUIBS), University of Las Palmas de Gran Canaria and Centro Hospitalario Universitario Insular Materno Infantil (CHUIMI), Canarian Health Service, Las Palmas de Gran Canaria, Spain; 21 Department of Preventive Medicine and Public Health, University of Granada, Granada, Spain; 22 Research Group on Community Nutrition and Oxidative Stress, University of Balearic Islands-IUNICS and IDISBA, Palma de Mallorca, Spain; 23 Institute of Biomedicine (IBIOMED), University of León, León, Spain; 24 Lipids and Vascular Risk Unit, Internal Medicine, Hospital Universitario de Bellvitge, Hospitalet de Llobregat,, Barcelona, Spain; 25 Division of Preventive Medicine, Faculty of Medicine, University of Jaén, Jaén, Spain; 26 Department of Endocrinology and Nutrition, Instituto de Investigación Sanitaria Hospital Clínico San Carlos (IdISSC), Madrid, Spain; 27 CIBER Diabetes Y Enfermedades Metabólicas (CIBERDEM), Instituto de Salud Carlos III (ISCIII), Madrid, Spain; 28 Department of Endocrinology, Institut d` Investigacions Biomédiques August Pi Sunyer (IDIBAPS), Hospital Clinic, University of Barcelona, Barcelona, Spain; 29 Lipid Clinic, Department of Endocrinology and Nutrition, Institut d’Investigacions Biomèdiques August Pi Sunyer (IDIBAPS), Hospital Clínic, Barcelona, Spain; 30 Department of Endocrinology and Nutrition, Hospital Fundación Jimenez Díaz, Instituto de Investigaciones Biomédicas IISFJD, University Autonoma, Madrid, Spain; 31 Nutritional Control of the Epigenome Group, IMDEA Food, CEI UAM + CSIC, Madrid, Spain; 32 Department of Nutrition, Harvard T.H. Chan School of Public Health, Boston, MA USA

**Keywords:** PREDIMED-Plus, Dietary change, Factors, Dietary adherence, Mediterranean diet, Randomized controlled trials

## Abstract

**Purpose:**

Long-term nutrition trials may fail to respond to their original hypotheses if participants do not comply with the intended dietary intervention. We aimed to identify baseline factors associated with successful dietary changes towards an energy-reduced Mediterranean diet (MedDiet) in the PREDIMED-Plus randomized trial.

**Methods:**

Longitudinal analysis of 2985 participants (Spanish overweight/obese older adults with metabolic syndrome) randomized to the active intervention arm of the PREDIMED-Plus trial. Dietary changes were assessed with a 17-item energy-reduced MedDiet questionnaire after 6 and 12 months of follow-up. Successful compliance was defined as dietary changes from baseline of ≥ 5 points for participants with baseline scores < 13 points or any increase if baseline score was ≥ 13 points. We conducted crude and adjusted multivariable logistic regression models to identify baseline factors related to compliance.

**Results:**

Consistent factors independently associated with successful dietary change at both 6 and 12 months were high baseline perceived self-efficacy in modifying diet (OR_6-month_: 1.51, 95% CI 1.25–1.83; OR_12-month_: 1.66, 95% CI 1.37–2.01), higher baseline fiber intake (OR_6-month_: 1.62, 95% CI 1.07–2.46; OR_12-month_: 1.62, 95% CI 1.07–2.45), having > 3 chronic conditions (OR_6-month_: 0.65, 95% CI 0.53–0.79; OR_12-month_: 0.76, 95% CI 0.62–0.93), and suffering depression (OR_6-month_: 0.80, 95% CI 0.64–0.99; OR_12-month_: 0.71, 95% CI 0.57–0.88).

**Conclusion:**

Our results suggested that recruitment of individuals with high perceived self-efficacy to dietary change, and those who initially follow diets relatively richer in fiber may lead to greater changes in nutritional recommendations. Participants with multiple chronic conditions, specifically depression, should receive specific tailored interventions.

**Trial registration:**

ISRCTN registry 89898870, 24th July 2014 retrospectively registered http://www.isrctn.com/ISRCTN89898870.

**Supplementary Information:**

The online version contains supplementary material available at 10.1007/s00394-021-02697-8.

## Introduction

Randomized controlled trials (RCTs) are considered the gold standard for clinical nutrition research. They offer scientific evidence of the highest quality level to infer causality of the health effects of diet interventions [1]. However, an important potential limitation in intervention trials occurs when participants do not comply with the intended dietary intervention, which may lead to worthless results [2].

In long-term randomized nutritional trials, participants require a high level of commitment to modify their diet. When participants do not sufficiently adherence to their assigned intervention, no substantial between-group contrast may be attained, and the magnitude of dietary effects can be considerably reduced. Consequently, the results of such trials render misleading results or null findings. As such, after 8 years of follow-up in the Randomized Controlled Dietary Modification Trial of the Women’s Health Initiative (WHI), a low-fat dietary intervention did not significantly reduce the risk of breast cancer, total cancer, coronary heart disease, certain chronic diseases, and total mortality when compared with a usual high-fat diet [3, 4]. However, the trial failed to achieve the 14% intended fat difference between the intervention and control groups; in fact, only 8% of energy fat reduction was achieved. In addition, further challenges to compliance with interventions on low-carbohydrate diets have been reported [5]. Lack of adherence was also reported in the Multiple Risk Factor Intervention Trial (MRFIT), an intervention that intended to obtain reductions in serum cholesterol (with diet), smoking cessation, and treatment of hypertension [6]. Contrasts in dietary changes between the control and intervention groups in the MRFIT were insufficient as to observe significant differences on cardiovascular disease (CVD) [7]. Even in the PREDIMED trial, which successfully demonstrated strong evidence on the role of the Mediterranean diet (MedDiet) on primary prevention of CVD [8], stronger beneficial effects would have been expected if participants had greater adhered to the intervention diet and the low-fat diet as reported in complementary per-protocol analyses. In fact, the per-protocol analyses suggested a much stronger risk reduction in the MedDiet groups compared to the control group than the intention-to-treat analysis.

Total exclusion in advance of overall non-compliant participants in dietary interventions is unattainable as well as it is unrealistic to believe that the original standard during RCTs can be maintained in normal life. Moreover, it is well known that RCTs usually attain only a suboptimal external validity. However, RCTs represent the gold standard for causal inference and recruiting a population that theoretically may help to maximize compliance with the targeted dietary interventions will make the trial more feasible and will potentially ensure sufficient exposure contrast, contributing to make the RCT more informative from a causal inference point of view. Otherwise, costly, long-term trials may continue failing to respond to its original hypothesis. Determining which patients’ and which design components maximize compliance can help investigators identify the most appropriate candidates and modifiable study features that are amenable to be redesigned. Limited knowledge exists on participants’ characteristics which may predict compliance to an intervention fostering the adherence to a healthy dietary pattern such as the MedDiet [9–15]. Most of this research has been limited to cross-sectional studies [11–15], with few longitudinal studies examining factors that predict dietary change in clinical trials [9, 10]. Thus, we aimed to identify factors of compliance to an energy-reduced MedDiet (erMedDiet) after 6 and 12 months of follow-up in the PREDIMED-Plus (PREvención con DIeta MEDiterránea Plus) randomized trial, a 6-year parallel-group, multicenter weight-loss lifestyle intervention program.

### Methods

#### Study design and participants

The present study is a longitudinal analysis restricted only to the intervention group of the PREDIMED-Plus trial. The study design and procedures of PREDIMED-Plus have been described in detail [16, 17]. In brief, it assesses the effect of an intensive lifestyle weight-loss intervention on the primary prevention of hard cardiovascular events. The intervention consists of an erMedDiet together with physical activity promotion and behavioral support for specific weight-loss goals on primary prevention of CVD events. More specifically, participants in the intervention group (*n* = 3406) regularly received individual motivational interviews and monthly phone calls, and attended group sessions in which trained dietitians encourage them to adopt suitable dietary and lifestyle changes. The erMedDiet intervention targeted a reduction of approximately 30% of estimated energy requirements, which represented a reduction goal of approximately 600 kcal/day [16, 17]. Moreover, the erMedDiet aimed to promote a better overall quality of the diet through the limitation of certain foods such as sugar-sweetened beverages, red and processed meats, butter and cream, added sugars, sweets and pastries, and refined cereals, including white bread, in favor of whole grains. Physical activity promotion included a face-to-face educational program [18] aimed to gradually increase participants’ aerobic physical activity levels to meet, at least, the World Health Organization (WHO) guidelines according to the participants’ age and health status [19]. Recommendations of physical activities also included static exercises to improve resistance, strength, flexibility, and balance. On the other hand, participants in the control group (*n* = 3468) were encouraged to follow an unrestricted energy MedDiet, had biannual educational sessions on the traditional MedDiet with ad libitum caloric intake, and received usual care of general lifestyle recommendations.

Potential candidates to participate in the PREDIMED-Plus trial were overweight/obese [body mass index (BMI) 27–40 kg/m^2^] males (aged 55–75 years of age) and females (60–75 years of age), with metabolic syndrome [20], and free of CVD at enrollment. The recruitment of participants of the PREDIMED-Plus took place from September 2013 until October 2016 in 23 Spanish centers. After completing a 4-week run-in period after the initial screening visit, participants were allocated in a 1:1 ratio (either the intervention or control arm) using a computer-generated random allocation sequence stratified by sex, age (< 65, 65–70, > 70 years of age), and center, which was concealed to principal investigators and staff members. Couples in the same household were randomly assigned as a unit. The Institutional Review Boards approved the study protocol of the recruiting centers participating in the study, and the PREDIMED-Plus trial was retrospectively registered at the International Standard Randomized Controlled Trial (ISRCTN 89898870; registration date, 24 July 2014), https://www.isrctn.com/ISRCTN89898870?q=ISRCTN89898870. All participants provided written informed consent.

### Outcome assessment

A 17-item erMedDiet questionnaire [16] was used to assess dietary adherence to the intervention group (Additional File 1: Table s1). The 17-point scale of erMedDiet adherence is an adapted version of the previously validated 14-item questionnaire used in the PREDIMED trial [21]. This modified version includes stricter cut-off points and additional items aimed to specifically capture the potential caloric restriction for existing weight-loss goals for the erMedDiet. Compliance with each of the 17 items of the questionnaire was scored with 1 point; otherwise, the score was 0 points. As such, the erMedDiet score ranged from 0 to 17, and the higher the score, the greater the adherence. Adherence to the erMedDiet was assessed by the PREDIMED-Plus trained dietitians at baseline and at each follow-up visit.

The outcome of the present study was to attain a *successful response* to the dietary intervention at 6- and 12-months of follow-up. Successful dietary response was defined as an increase in at least 5 points from baseline to follow-up in the erMedDiet score or any positive increase (≥ 1 point) for participants with 13 or higher scores at baseline. Participants, therefore, were classified as *adherent* and *non-adherent* based on their 17-item erMedDiet score change from baseline to 6-month and from baseline to 12-month follow-up visits (Additional File 1: Fig. s1).

### Covariate assessment

Usual diet was ascertained at baseline and follow-up visits by trained dietitians throughout face-to-face interviews using the Spanish version of a previously validated 143-item semiquantitative food-frequency questionnaire (SFFQ) [22, 23]. Food consumption frequencies were registered in nine categories ranging from “never or seldom” to “≥ 6 times/day” and food composition tables were used to calculate energy and nutrient intakes for each participant [24, 25]. An additional questionnaire was used to collect updated information in each visit about socio-demographics, personal and family history of disease, and lifestyles, including leisure-time physical activity, assessed by the Rapid Assessment of Physical Activity Questionnaires (RAPA-1 and RAPA-2) [26], the validated Minnesota-REGICOR short physical activity questionnaire [27], and the validated Spanish version of the Nurses’ Health Study questionnaire [28]. Weight and height were measured by registered dietitians with standardized procedures. Blood pressure was measured in triplicate by registered nurses using a validated semiautomatic oscillometer (Omron HEM 297 705C). Blood samples were collected after an overnight fast to determine levels of fasting blood glucose, among other determinations, with standard enzymatic methods.

### Independent factors

Potential baseline factors of compliance to MedDiet were selected considering existing literature and preceding results of the PREDIMED trial [9, 10]. We categorized candidate factors in the following groups: socio-demographics, health-related characteristics, study design features, lifestyle behaviors, and baseline energy and nutrient intake. *Sociodemographic characteristics* included sex, age (< 65 years, and ≥ 65 years), marital status (married, single, widowed, and others/missing), highest attained educational level (college/university, secondary, primary or less), occupation (retired, working, unemployed/unable to work, housewife), and number of people living in the household (continuous). *Health-related characteristics* comprised family history of premature CVD (dichotomous), number of chronic conditions (≤ 3 and > 3), self-reported score of nervousness and/or aggressiveness behavior (quartiles), body weight (continuous, per 5 kg), waist circumference (continuous, per 5 cm), systolic and diastolic blood pressure (both continuous, per 5 mm Hg), and fasting blood glucose (continuous, per 10 mg/dL). Family history of premature CVD was defined as any immediate family member deceased by CVD younger than 55 years for men, and 65 years for women; for number of chronic conditions the following diagnoses were considered: hypertension, obesity (BMI ≥ 30 kg/m^2^), type 2 diabetes, hypercholesterolemia, cancer, and depression; self-reported measures of nervousness and/or aggressiveness behavior were self-reported on a scale from 1 (very low self-perception) to 10 (very high self-perception). *Study design features* included recruitment period (< 1st year, between 1st and 2nd year, between 2nd and 3rd year, and after 3rd year of recruitment), and total field center workload (below and above the median). Recruitment year was referred to the period (years) in which participants were recruited, from the date of the first recruited participant (Sep/05/2013) to the date of the last recruited participant (Oct/31/2016); total field center workload was quantified as the number of participants per center. *Lifestyle behavior* included leisure-time physical activity (METs-min/week, quartiles) and RAPA test (sedentary/under-active, under-active regular-activities, under-active regular, and active), 30-s chair test (number of repeats, quartiles), smoking status (never, current, former), alcohol intake other than wine (≤ 5 g/day and > 5 g/day), sleeping (hours, quartiles), and self-efficacy for diet modification (little/some and high). Self-efficacy for diet modification was defined as participants’ beliefs in their ability to achieve dietary change with three options (little, some, or high); given the scarce number of participants responding “little”, we merged the two lower categories. *Energy and nutrient intake* factors comprised baseline total energy intake (kcal/day, sex-specific quartiles), predefined limits of energy intake (within limits: 500–3500 kcal for women and 800–4000 for men, and beyond limits: < 500 or > 3500 kcal/day for women and < 800 or > 4000 kcal/day for men) were used to select the analytical sample [29], fruit + vegetable consumption (g/day, sex-specific quartiles), meat consumption (g/day, sex-specific quartiles), baseline dietary fat intake (%E, quartiles), fiber intake (g/day, sex-specific quartiles), carbohydrate quality index (CQI) (score, quartiles), and baseline 17-point erMedDiet adherence (score, quartiles). CQI referred to the quality of dietary carbohydrate intake and was constructed upon the following four carbohydrate quality domains: high total dietary fiber intake, low glycemic index, high whole-grain carbohydrate: total grain carbohydrate ratio, and high solid carbohydrate: total carbohydrate ratio [30].

### Statistical analysis

Descriptive statistics, including means and standard deviations (SD) for quantitative variables and percentages for categorical variables, were used to describe baseline characteristics of participants categorized as *non-adherent* and *adherent* according to their 17-item erMedDiet score change from baseline to 6 and 12 months of follow-up. Chi-squared tests for categorical variables and Student’s *t* test (the assumption of normality was not violated given the large sample size) for continuous variables were used to assess differences between groups. We performed crude and adjusted multivariable logistic regression models to evaluate the probability of appropriate compliance according to the aforementioned baseline factors. Hence, odds ratios (OR) < 1 suggest *poor compliance* of dietary change, whereas ORs > 1 suggest *successful compliance*. For categorical variables, we used as reference the category which was expected a priori to show a greater odds of compliance, while the reference category for ordinal factors was considered the lowest category (usually, the first quartile). Tests of linear trend across categories of potential factors were run assigning the median to each category and treating the resulting variables as continuous. Participants with missing values in candidate factors were categorized as a separate group.

We conducted several sensitivity analyses using multivariable logistic regression models to corroborate the consistency of factors under different scenarios: excluding participants with any missing value; excluding participants with baseline score ≥ 13 points in the 17-item erMedDiet adherence questionnaire; and using an alternative definition of the outcome, especifically, scoring > 12 points at follow-up (instead of our original definition, Additional File 1: Fig. s1).

All analyses were performed using Stata software, version 16.0 (StataCorp LP) using the PREDIMED-Plus database updated in March 2019. A two-sided *p* value < 0.05 was deemed as statistically significant.

## Results

### Sample characteristics

**Fig. 1:**
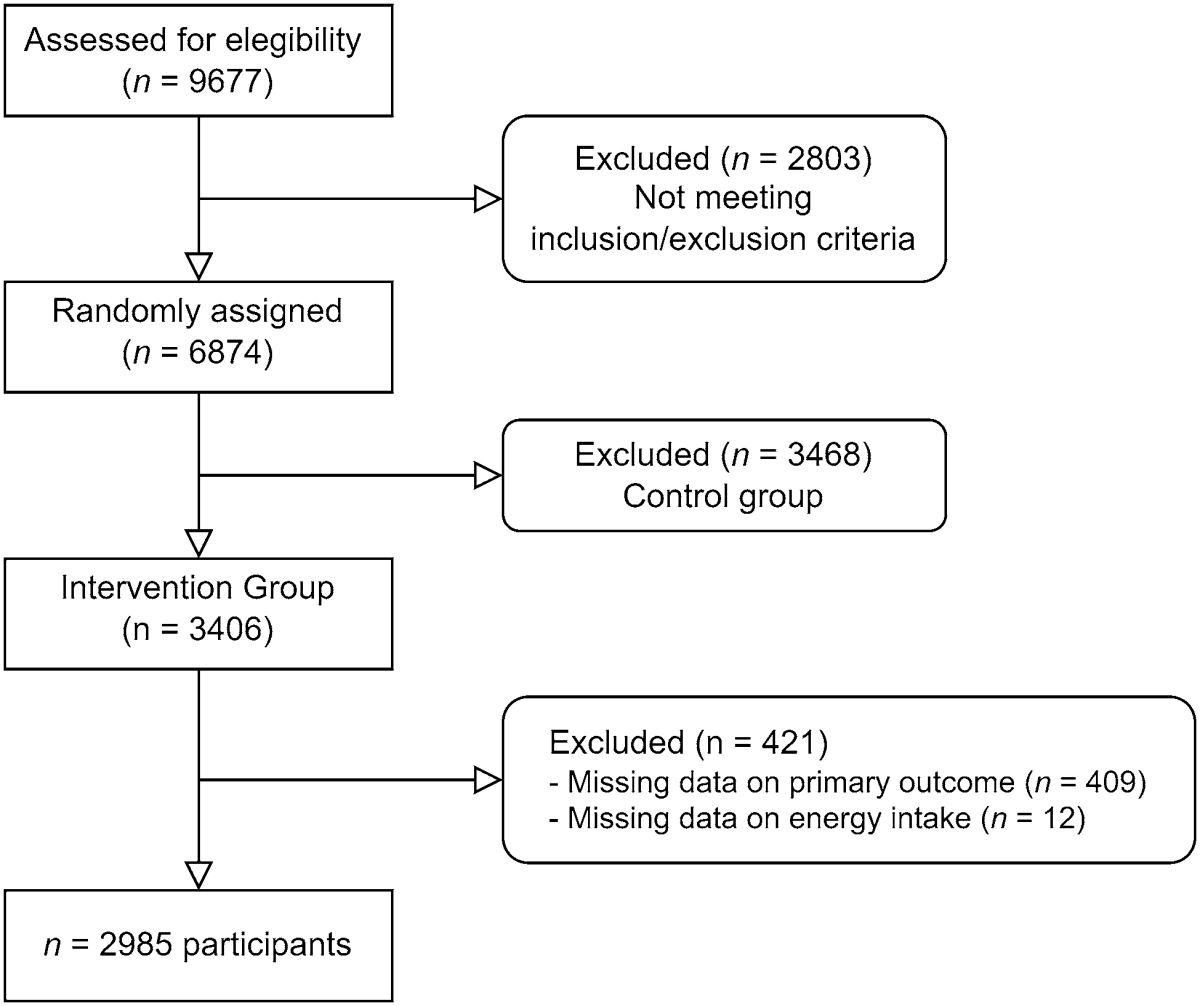
Flow chart of the participants of the study. The PREDIMED-Plus trial

We excluded participants in the control group of the trial. Among the 3406 participants of the intervention arm of the PREDIMED-Plus trial, we excluded 409 (12%) individuals with missing data on the 17-item erMedDiet questionnaire either at baseline or during follow-up, and 12 (0.4%) participants with missing information of energy intake. The remaining 2985 individuals (1445 females and 1540 males) were included in our analyses. There were neither withdrawals nor losses to follow-up before the study completion (Fig. 1).

The mean (SD) age of the 2985 participants included in the study was 64.9 (4.9) years, 76% of them were married, 56% retired, 47% received primary education or less, and 45% never smoked. Baseline characteristics of participants according to their changes in the adherence to the 17-item erMedDiet from baseline to 6-month and 12-month follow-up period are shown in Table 1. At 6 months, nearly half of the participants (49.5%) successfully attained an adequate change, while the proportion of adherent participants at 12 months was slightly higher (52.4%).

**Table 1:** Baseline characteristics of the intervention group in the PREDIMED-Plus trial (*n* = 2,985) according to attained 6-month and 12-month adherence to a 17-item energy-reduced MedDiet score

Baseline characteristics	6-month follow-up			12-month follow-up		
	Non-adherent^1^ (decreasing, equal or increment < 5 points) (*n* = 1507)	Adherent^1^ (increasing ≥ 5 points if baseline < 13p or any increase if baseline ≥ 13p) (*n* = 1478)	*p* value	Non-adherent^1^ (decreasing, equal or increment < 5 points) (*n* = 1422)	Adherent^1^ (increasing ≥ 5 points if baseline < 13p or any increase if baseline ≥ 13p) (*n* = 1563)	*p* value
Socio-demographics						
Sex, women	50.5	46.3	0.021	51.1	45.9	0.005
Age, years, mean (SD)	65.2 (4.9)	64.7 (5.0)	0.005	65.2 (5.0)	64.7 (4.9)	0.016
Marital status			0.034			0.020
Married	73.8	77.2		73.1	77.6	
Single	6.4	4.3		6.0	4.8	
Widowed	10.9	10.9		11.3	10.5	
Others/misisng	9.0	7.6		9.6	7.1	
Attained education level			0.621			0.725
College/university	22.2	20.8		22.1	21.0	
Secondary	30.7	30.5		31.1	30.1	
Primary or less	46.4	47.5		45.9	47.9	
Occupation			0.657			0.833
Retired	55.9	55.6		55.0	56.5	
Working	20.1	22.0		21.0	21.1	
Unemployed or unable to work	8.0	7.9		8.2	7.7	
Housewife	15.3	13.9		15.1	14.1	
Number of people in household, mean (SD)	1.4 (1.1)	1.4 (1.2)	0.351	1.4 (1.1)	1.4 (1.0)	0.069
Baseline health-related characteristics						
Hypertension	84.4	82.9	0.260	83.5	83.8	0.802
Obesity	72.7	72.7	0.965	71.9	73.4	0.334
Type 2 diabetes	32.1	23.5	< 0.001	31.3	24.7	< 0.001
Hypercholesterolemia	71.1	68.7	0.154	70.1	69.8	0.853
^2^Family history of premature CVD	12.3	13.1	0.557	12.9	12.5	0.787
Cancer	7.6	7.1	0.582	7.5	7.3	0.867
Depression	21.0	17.6	0.017	21.9	17.0	0.001
^3^Average number of chronic conditions, mean (SD)	2.9 (1.0)	2.7 (1.0)	< 0.001	2.9 (1.0)	2.8 (1.0)	0.007
^4^Self-reported measure of nervousness and/or aggressiveness behavior			0.717			0.219
Q1 (low)	27.3	27.4		28.4	26.4	
Q2	37.6	35.8		37.5	36.0	
Q3	11.5	11.8		11.3	11.9	
Q4 (high)	23.6	25.0		22.8	25.7	
Body weight, kg, mean (SD)	85.8 (12.9)	87.2 (13.1)	0.003	85.9 (13.3)	87.0 (12.8)	0.023
Waist circumference, cm, mean (SD)	107 (10)	108 (10)	0.013	107 (10)	108 (10)	0.213
BMI, kg/m^2^, mean (SD)	32.5 (3.4)	32.6 (3.5)	0.299	32.5 (3.5)	32.6 (3.4)	0.541
SBP, mmHg, mean (SD)	139 (18)	140 (17)	0.014	139 (17)	140 (17)	0.191
DBP, mmHg, mean (SD)	80.5 (9.9)	81.1 (9.9)	0.096	80.7 (10.2)	81 (9.7)	0.464
Fasting blood glucose, mg/dL, mean (SD)	113 (28)	112 (26)	0.197	113 (28)	112 (27)	0.249
Study design features						
^5^Recruitment year			0.005			< 0.001
< 1st	10.6	7.4		11.3	6.9	
1st–2nd	25.4	25.6		24.0	26.9	
2nd–3rd	48.0	52.9		49.0	51.8	
> 3rd	16.1	14.1		15.8	14.4	
^6^Total workload of center, participants, mean (SD)	151 (64)	154 (62)	0.330	152 (64)	153 (62)	0.547
Baseline lifestyles and behaviors						
Physical activity						
METs-min/week, mean (SD)	2498 (2312)	2332 (2170)	0.043	2398 (2180)	2431 (2301)	0.687
RAPA test			0.278			0.197
Level 1 (sedentary or under-active)	18.4	18.8		19.5	17.9	
Level 2 (under-active regular—light activities)	35.3	37.0		36.8	35.6	
Level 3 (under-active regular)	17.3	18.3		16.3	19.1	
Level 4 (active)	29.0	25.8		27.4	27.4	
Chair test 30 s, repeats, mean (SD)	13.1 (4.9)	13.4 (4.7)	0.140	13.1 (4.9)	13.4 (4.7)	0.053
Smoking status, n (%)			0.574			0.469
Never smokers	44.3	45.3		44.4	45.1	
Current smokers	13.9	12.7		14.2	12.5	
Former smokers	41.2	41.7		40.8	42.0	
Alcohol intake other than wine, g/day, mean (SD)	4.5 (8.6)	4.7 (8.3)	0.272	4.5 (9.0)	4.6 (8.0)	0.210
Sleeping, hours/day, mean (SD)	7.0 (1.2)	7.0 (1.2)	0.644	7.0 (1.2)	7.0 (1.2)	0.960
High perceived self-efficacy for diet modification	72.9	77.7	0.003	71.9	78.3	< 0.001
Baseline dietary pattern, total energy, and nutrient intake						
Baseline 17-item energy-reduced MedDiet score, mean (SD)	9.4 (2.3)	7.5 (2.6)	< 0.001	9.5 (2.3)	7.5 (2.6)	< 0.001
Total energy intake, (kcal/day), mean (SD)	2365 (610)	2438 (588)	0.001	2356 (599)	2443 (598)	< 0.001
Participants with total energy intake beyond predefined limits (Willett)	2.5	2.4	0.877	2.2	2.6	0.431
Baseline fruit + vegetable consumption, g/day, mean (SD)	714 (293)	676 (266)	< 0.001	716 (290)	677 (270)	< 0.001
Baseline meat consumption, g/day, mean (SD)	146 (60)	152 (59)	0.003	146 (60)	151 (59)	0.008
Baseline dietary fat intake, % E, mean (SD)	39.4 (6.8)	39.6 (6.3)	0.352	39.7 (6.7)	39.4 (6.4)	0.251
Baseline fiber intake, g/day, mean (SD)	27.2 (9.4)	26.1 (8.7)	< 0.001	27.2 (9.5)	26.1 (8.7)	< 0.001
Baseline carbohydrate Quality Index^7^, mean (SD)	10.1 (2.6)	9.6 (2.6)	< 0.001	10.2 (2.5)	9.6 (2.6)	< 0.001

Values are percentages of participants unless otherwise indicated.

*BMI* body mass index, *CC* chronic conditions, *CVD* cardiovascular disease, *DBP* diastolic blood pressure, *E* energy, *MedDiet* Mediterranean diet, *MET* metabolic equivalent, *Q* quartile, *RAPA* rapid assessment of physical activity, *SBP* systolic blood pressure

Data available in the intervention group of the PREDIMED-Plus trial (*n* = 2985); for marital status (*n* = 10 missing); for attained education level (*n* = 29 missing); for occupation (*n* = 20 missing); for number of people in household (*n* = 5 missing); for self-reported measure of nervousness and/or aggressiveness behavior (*n* = 26 missing); for SBP (*n* = 23 missing); for DBP (*n* = 23 missing); for fasting blood glucose (*n* = 42 missing); for RAPA test (*n* = 1 missing); for smoking status (*n* = 14 missing); for sleeping hours (*n* = 38 missing).

^1^Adherence to Mediterranean diet was evaluated using a 17-point scale of adherence to an energy-reduced MedDiet questionnaire (1 point for each item). Participants with an increase of ≥ 5 points from baseline to follow-up were classified in the “*adherent group*”. Participants with ≥ 13 points at baseline and any positive increase (≥ 1 point) from baseline to follow-up were additionally classified in the “*adherent group*”. Detailed information is provided in Additional File 1: Figure s1.

^2^Family history of premature CVD was defined as any immediate family member deceased younger than 55 years for men and 65 years for women.

^3^Number of chronic conditions was calculated by summing the following chronic conditions (1 point for each condition): hypertension, obesity, type 2 diabetes, hypercholesterolemia, cancer, and depression).

^4^Self-reported measure of nervousness and/or aggressiveness behavior was reported on a scale from 1 (very low self-perception) to 10 (very high self-perception).

^5^Recruitment year was referred to the period (years) in which participants were recruited, from the date of the first recruited participant to the date of the last recruited participant (< 1, 1–2, 2–3, and > 3 years).

^6^Total workload of center was measured as the number of participants in the intervention group per center

^7^Carbohydrate Quality Index was referred to the quality of dietary carbohydrate intake (high total dietary fiber intake, low glycemic index, high whole-grain carbohydrate: total grain carbohydrate ratio, and high solid carbohydrate: total carbohydrate ratio.

### Factors of dietary change (6 months)

**Fig. 2:**
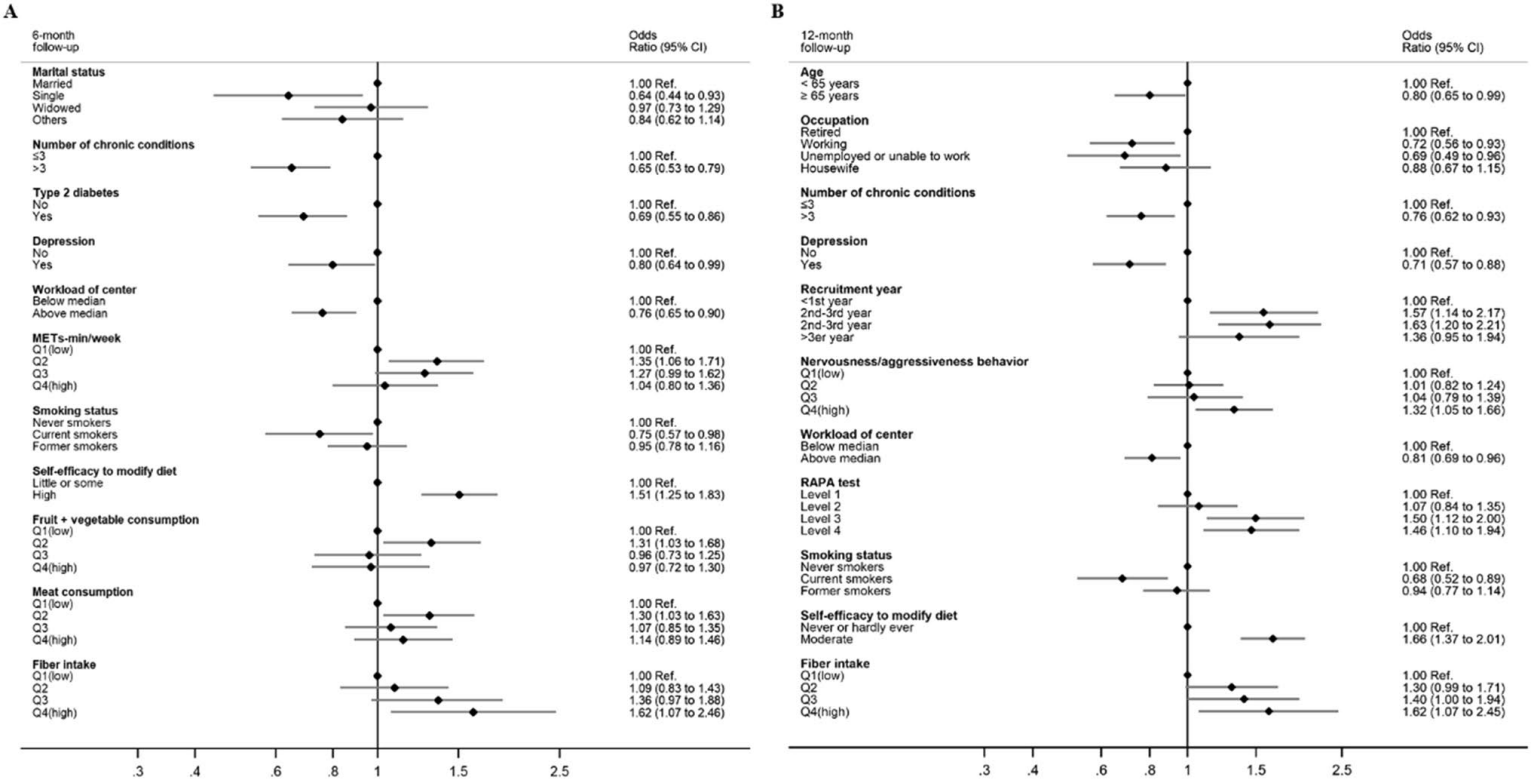
Independent factors of good adherence. Odds ratios (OR) and 95% confidence intervals (95% CI) of good adherence (increasing ≥ 5 points if baseline < 13 or any increase if baseline ≥ 13) to the MedDiet intervention at **A** 6 months and **B** 12 months of follow-up in the active intervention group of the PREDIMED-Plus trial (*n* = 2,985). Adherence to Mediterranean diet was evaluated using a 17-point scale of adherence to an energy-reduced MedDiet questionnaire (1 point for each item). Participants with an increase of ≥ 5 points from baseline to follow-up were classified in the “*adherent group*”. Participants with ≥ 13 points at baseline and positive increase (≥ 1 point) from baseline to follow-up were additionally classified in the “*adherent group*”. Detailed information is provided in Additional File 1: Figure s1. Dietary fat intake was expressed as % of energy. Fiber intake was expressed in g/day. Recruitment year was referred to the period (years) in which participants were recruited, from the date of the first recruited participant (9/05/2013) to the date of the last recruited participant (10/31/2016). Nervousness/aggressiveness behavior was self-reported on a scale from 1 (very low self-perception) to 10 (very high self-perception). RAPA test levels were categorized as: level 1 (sedentary or under-active), level 2 (under-active regular – light activities), level 3 (under-active regular), and level 4 (active). Workload of center was measured as total number of persons-years of follow-up

Table 2 shows the main results for the crude and multivariable logistic regression analyses for the association between baseline characteristics and dietary compliance after 6 and 12 months of follow-up in the intervention group of the trial. Baseline characteristics significantly associated with *better* compliance at 6-month follow-up in multivariable analyses were: moderate level of physical activity (METs-min/week), high self-reported self-efficacy at baseline to change their diet, moderate consumption of fruit + vegetables, moderate meat consumption, and high fiber intake. On the other hand, being single (vs. married), having more than three chronic conditions, and being a current smoker (vs. never smoker) were associated with *poorer* compliance. Regarding study design features, high total field center workload was the only predictor associated with *poorer* compliance. Additionally, participants with a previous diagnosis of type 2 diabetes and depression were less likely to adhere to the intervention (Table 3). Noteworthy, when introducing in the model the predictor *chronic conditions* categorized by the number of conditions (≤ 1, 2, 3, 4, and ≥ 5) instead of as a dichotomous variable (≤ 3 and > 3 conditions), the odds of compliance monotonically decreased as the number of chronic conditions increased (OR: 0.77, 95% CI 0.56, 1,04 for 2 conditions; OR: 0.75, 95% CI 0.55, 1,01 for 3 conditions; OR: 0.52, 95% CI 0.37, 0.73 for 4 conditions; OR: 0.41, 95% CI 0.25, 0,68 for ≥ 5 conditions; ref.: ≤ 1 conditions; data not shown). Additionally, the likelihood of successful compliance decreased across successive quartiles of higher baseline adherence to the erMedDiet score, probably representing a ceiling effect (Additional File 1: Table s2). The factors independently associated with dietary compliance at 6-month follow-up are shown in Fig. 2.

**Table 2:** Odds ratios (OR) and 95% confidence intervals (95% CI) of attaining good adherence^1^ (increasing ≥ 5 points if baseline < 13p or *any* increase if baseline ≥ 13p) to the MedDiet intervention at 6 and 12 months of follow-up in the active intervention group of the PREDIMED-Plus trial (*n* = 2,985)

Baseline characteristics	*n*	OR (95% CI) for adherence (increasing ≥ 5 points if baseline < 13p or any increase if baseline ≥ 13p)^1^ to the MedDiet intervention (adherent vs. non-adherent) ^2^							
		6 month-follow-up				12 month-follow-up			
		Crude^3^	*p* value	Multivariable^4^	*p* value	Crude^3^	*p* value	Multivariable^4^	*p* value
Socio-demographics									
Sex									
Men	1540	1.00 (ref)	–	1.00 (ref)	–	1.00 (ref)	–	1.00 (ref)	–
Women	1445	0.84 (0.73–0.97)	0.021	1.19 (0.93–1.52)	0.173	0.81 (0.70–0.94)	0.005	1.10 (0.86–1.40)	0.470
Age, years									
< 65	1404	1.00 (ref)	–	1.00 (ref)	–	1.00 (ref)	–	1.00 (ref)	–
≥ 65	1581	0.88 (0.77–1.02)	0.094	0.86 (0.70–1.07)	0.171	0.89 (0.77–1.03)	0.109	**0.80 (0.65–0.99)**	**0.039**
Marital status									
Married	2253	1.00 (ref)	–	1.00 (ref)	–	1.00 (ref)	–	1.00 (ref)	–
Single	160	0.65 (0.47–0.90)	0.010	**0.64 (0.44–0.93)**	**0.020**	0.76 (0.55–1.04)	0.089	0.83 (0.57–1.20)	0.317
Widowed	325	0.96 (0.76–1.21)	0.710	0.97 (0.73–1.29)	0.837	0.87 (0.69–1.10)	0.254	0.92 (0.69–1.22)	0.544
Others/missing	247	0.81 (0.62–1.05)	0.114	0.84 (0.62–1.14)	0.253	0.70 (0.54–0.91)	0.008	0.75 (0.55–1.02)	0.066
Attained education level									
College/university	642	1.00 (ref)	–	1.00 (ref)		1.00 (ref)	–	1.00 (ref)	–
Secondary	913	1.06 (0.86–1.30)	0.581	0.94 (0.75–1.18)	0.602	1.02 (0.83–1.25)	0.847	0.85 (0.67–1.07)	0.164
Primary or less	1401	1.09 (0.90–1.31)	0.371	1.06 (0.84–1.33)	0.641	1.10 (0.91–1.33)	0.319	1.01 (0.80–1.27)	0.938
Missing	29	1.54 (0.72–3.27)	0.265	1.28 (0.56–2.96)	0.558	1.03 (0.49–2.16)	0.947	0.80 (0.35–1.85)	0.602
Occupation									
Retired	1665	1.00 (ref)	–	1.00 (ref)	–	1.00 (ref)	–	1.00 (ref)	–
Working	628	1.10 (0.92–1.32)	0.309	0.88 (0.68–1.14)	0.321	0.98 (0.82–1.18)	0.836	**0.72 (0.56–0.93)**	**0.013**
Unemployed or unable to work	237	1.00 (0.76–1.31)	0.999	0.81 (0.58–1.14)	0.226	0.92 (0.70–1.21)	0.568	**0.69 (0.49–0.96)**	**0.029**
Housewife	435	0.91 (0.74–1.13)	0.405	0.85 (0.65–1.11)	0.228	0.91 (0.73–1.12)	0.361	0.88 (0.67–1.15)	0.363
Missing	20	0.84 (0.35–2.04)	0.698	0.99 (0.36–2.70)	0.984	0.72 (0.30–1.76)	0.476	0.88 (0.32–2.39)	0.799
Number of people in household (continuous)	2985	1.03 (0.96–1.11)	0.344	0.96 (0.88–1.05)	0.387	1.07 (0.99–1.14)	0.070	1.00 (0.92–1.09)	0.943
Health-related characteristics									
^5^Family history of premature CVD									
No	2606	1.00 (ref)	–	1.00 (ref)	–	1.00 (ref)	–	1.00 (ref)	–
Yes	379	1.07 (0.86–1.32)	0.557	1.09 (0.86–1.39)	0.476	0.97 (0.78–1.20)	0.787	0.94 (0.74–1.20)	0.629
^6^Number of chronic conditions									
≤ 3	2267	1.00 (ref)	–	1.00 (ref)	–	1.00 (ref)	–	1.00 (ref)	–
> 3	718	0.67 (0.56–0.79)	< 0.001	**0.65 (0.53–0.79)**	** < 0.001**	0.72 (0.61–0.86)	< 0.001	**0.76 (0.62–0.93)**	**0.009**
^7,8^Self-reported measure of nervousness and/or aggressiveness behavior, score									
Q1(< 4)	816	1.00 (ref)	–	1.00 (ref)	–	1.00 (ref)	–	1.00 (ref)	–
Q2 (4–5)	1096	0.95 (0.79–1.13)		0.89 (0.72–1.09)		1.04 (0.86–1.24)		1.01 (0.82–1.24)	
Q3 (6)	347	1.02 (0.79–1.31)		0.92 (0.69–1.21)		1.13 (0.88–1.46)		1.04 (0.79–1.39)	
Q4 (> 6)	726	1.05 (0.86–1.29)	0.691	1.10 (0.88–1.38)	0.696	1.22 (1.00–1.49)	0.066	**1.32 (1.05–1.66)**	**0.045**
Body weight, (per 5 kg)	2985	1.04 (1.01–1.07)	0.003	1.02 (0.96–1.08)	0.522	1.03 (1.01–1.06)	0.023	1.03 (0.97–1.09)	0.328
Waist circumference (per 5 cm)	2985	1.05 (1.01–1.09)	0.014	1.02 (0.94–1.09)	0.681	1.02 (0.99–1.06)	0.213	0.97 (0.90–1.05)	0.476
SBP (per 5 mm Hg)	2985	1.03 (1.01–1.05)	0.013	1.02 (1.00–1.06)	0.098	1.01 (0.99–1.04)	0.190	1.00 (0.98–1.03)	0.766
DBP (per 5 mm Hg)	2985	1.03 (0.99–1.07)	0.100	1.02 (0.97–1.07)	0.414	1.01 (0.98–1.05)	0.487	1.01 (0.96–1.07)	0.567
Fasting blood glucose (per 10 mg/dL)	2985	0.98 (0.96–1.01)	0.147	1.00 (0.97–1.03)	0.900	0.98 (0.96–1.01)	0.223	1.00 (0.97–1.03)	0.864
Study design features									
^9^Recruitment year									
< 1st	268	1.00 (ref)	–	1.00 (ref)	–	1.00 (ref)	–	1.00 (ref)	–
1st–2nd	761	1.45 (1.09–1.92)	0.010	1.11 (0.81–1.53)	0.510	1.82 (1.38–2.42)	< 0.001	**1.57 (1.14–2.17)**	**0.005**
2nd–3rd	1506	1.57 (1.20–2.04)	0.001	1.28 (0.94–1.74)	0.112	1.73 (1.33–2.25)	< 0.001	**1.63 (1.20–2.21)**	**0.002**
> 3rd	450	1.28 (0.95–1.74)	0.109	0.98 (0.69–1.40)	0.919	1.48 (1.09–2.01)	0.012	1.36 (0.95–1.94)	0.088
^10^Total workload of center, participants in intervention group									
Below median (* n* ≤ 128)	1498	1.00 (ref)	–	1.00 (ref)	–	1.00 (ref)	–	1.00 (ref)	–
Above median (*n* > 128)	1487	0.81 (0.70–0.93)	0.003	**0.76 (0.65–0.90)**	**0.002**	0.83 (0.72–0.96)	0.014	**0.81 (0.69–0.96)**	**0.016**
Lifestyle behavior									
Physical activity									
^8^METs-min/week									
Q1 (< 840)	778	1.00 (ref)	–	1.00 (ref)	–	1.00 (ref)	–	1.00 (ref)	–
Q2 (840–1818)	720	1.22 (0.99–1.49)		**1.35 (1.06–1.71)**		1.01 (0.82–1.23)		0.99 (0.78–1.26)	
Q3 (1819–3356)	762	1.09 (0.89–1.33)		1.27 (0.99–1.62)		1.01 (0.82–1.23)		1.05 (0.82–1.34)	
Q4 (> 3356)	725	0.88 (0.72–1.08)	0.057	1.04 (0.80–1.36)	0.619	1.04 (0.85–1.27)	0.717	1.10 (0.84–1.43)	0.412
RAPA test									
Level 1 (sedentary or under-active)	556	1.00 (ref)	–	1.00 (ref)	–	1.00 (ref)	–	1.00 (ref)	–
Level 2 (under-active regular—light activities)	1079	1.03 (0.84–1.26)	0.790	1.01 (0.80–1.28)	0.919	1.06 (0.86–1.30)	0.605	1.07 (0.84–1.35)	0.579
Level 3 (under-active regular)	531	1.04 (0.82–1.32)	0.733	1.12 (0.84–1.49)	0.436	1.28 (1.01–1.62)	0.043	**1.50 (1.12–2.00)**	**0.006**
Level 4 (active)	819	0.87 (0.70–1.08)	0.221	1.13 (0.86–1.50)	0.378	1.09 (0.88–1.35)	0.423	**1.46 (1.10–1.94)**	**0.008**
^8^Chair test 30 s, repeats									
Q1 (< 12)	962	1.00 (ref)	–	1.00 (ref)	–	1.00 (ref)	–	1.00 (ref)	–
Q2 (12–13)	665	1.17 (0.96–1.43)		1.06 (0.84–1.32)		1.13 (0.93–1.38)		0.99 (0.79–1.24)	
Q3 (14–16)	762	1.07 (0.89–1.30)		1.01 (0.81–1.26)		1.14 (0.94–1.38)		1.04 (0.83–1.30)	
Q4 (> 16)	596	1.10 (0.89–1.34)	0.552	0.94 (0.73–1.20)	0.563	1.11 (0.91–1.37)	0.301	0.89 (0.69–1.14)	0.440
Smoking status, *n* (%)									
Never smokers	1337	1.00 (ref)	–	1.00 (ref)	–	1.00 (ref)	–	1.00 (ref)	–
Current smokers	397	0.90 (0.72–1.12)	0.348	**0.75 (0.57–0.98)**	**0.038**	0.87 (0.69–1.08)	0.206	**0.68 (0.52–0.89)**	**0.005**
Former smokers	1237	0.99 (0.85–1.16)	0.903	0.95 (0.78–1.16)	0.634	1.02 (0.87–1.19)	0.846	0.94 (0.77–1.14)	0.533
Missing	14	0.55 (0.18–1.66)	0.293	0.42 (0.13–1.40)	0.159	0.67 (0.23–1.95)	0.465	0.58 (0.18–1.88)	0.361
Alcohol intake other than wine									
Never	997	1.00 (ref)	–	1.00 (ref)	–	1.00 (ref)	–	1.00 (ref)	–
≤ 5 g/day	1266	1.05 (0.89–1.23)	0.603	0.93 (0.76–1.12)	0.432	1.07 (0.91–1.27)	0.411	0.95 (0.78–1.16)	0.619
> 5 g/day	722	1.12 (0.93–1.36)	0.235	0.95 (0.74–1.22)	0.683	1.19 (0.99–1.45)	0.069	1.00 (0.78–1.28)	0.993
^8^Sleeping, hours/day									
Q1 (< 7)	964	1.00 (ref)	–	1.00 (ref)	–	1.00 (ref)	–	1.00 (ref)	–
Q2 (7)	984	0.99 (0.83–1.18)		0.98 (0.80–1.20)		0.94 (0.79–1.12)		0.91 (0.75–1.12)	
Q3 (8)	777	0.87 (0.72-.1.05)		0.86 (0.70–1.06)		0.99 (0.82–1.20)		1.00 (0.81–1.24)	
Q4 (> 8)	260	1.14 (0.87–1.50)	0.763	1.10 (0.80–1.50)	0.634	1.11 (0.85–1.47)	0.602	1.00 (0.74–1.37)	0.910
Self-efficacy for diet modification									
Little or some	738	1.00 (ref)	–	1.00 (ref)	–	1.00 (ref)	–	1.00 (ref)	–
High	2247	1.29 (1.09–1.53)	0.003	**1.51 (1.25–1.83)**	** < 0.001**	1.41 (1.19–1.66)	< 0.001	**1.66 (1.37–2.01)**	** < 0.001**
Total energy and nutrient intake									
^8^Total energy intake, kcal/day									
Q1 (men < 2121; women < 1889)	747	1.00 (ref)	–	1.00 (ref)	–	1.00 (ref)	–	1.00 (ref)	–
Q2 (men 2121–2477; women: 1889–2214)	746	1.20 (0.98–1.47)		1.00 (0.78–1.27)		1.15 (0.94–1.40)		0.99 (0.78–1.27)	
Q3 (men 2478–2885; women: 2215–2564)	746	1.41 (1.15–1.73)		1.02 (0.79–1.32)		1.43 (1.17–1.75)		1.10 (0.84–1.42)	
Q4 (men > 2885; women: > 2564)	746	1.36 (1.11–1.67)	0.002	0.84 (0.62–1.13)	0.234	1.35 (1.11–1.66)	0.001	0.88 (0.66–1.19)	0.464
Predefined limits of energy intake (Willet), kcal/day									
Within limits (men 800–4000; women 500–3500)	2913	1.00 (ref)		1.00 (ref)	–	1.00 (ref)	–	1.00 (ref)	–
Beyond limits (men < 800 or > 4000; women < 500 or > 3500)	72	0.96 (0.60–1.54)	0.877	0.67 (0.39–1.17)	0.157	1.21 (0.75–1.94)	0.431	1.01 (0.58–1.75)	0.985
^8^Fruit + vegetable consumption, g/day									
Q1 (men, < 473; women < 544)	747	1.00 (ref)	–	1.00 (ref)	–	1.00 (ref)	–	1.00 (ref)	–
Q2 (men 473–624; women 544–698)	746	1.13 (0.93–1.39)		**1.31 (1.03–1.68)**		0.92 (0.75–1.13)		0.96 (0.75–1.23)	
Q3 (men 625–795; women 699–886)	746	0.84 (0.69–1.03)		0.96 (0.73–1.25)		0.86 (0.70–1.06)		0.91 (0.70–1.20)	
Q4 (men > 795; women > 886)	746	0.80 (0.66–0.99)	0.004	0.97 (0.72–1.30)	0.417	0.73 (0.60–0.90)	0.002	0.82 (0.61–1.10)	0.171
^8^Meat consumption, g/day									
Q1 (men < 114; women < 105)	747	1.00 (ref)	–	1.00 (ref)	–	1.00 (ref)	–	1.00 (ref)	–
Q2 (men 114–147; women 105–137)	746	1.30 (1.06–1.59)		**1.30 (1.03–1.63)**		1.15 (0.94–1.41)		1.10 (0.87–1.38)	
Q3 (men 148–188; women 138–174)	747	1.19 (0.97–1.46)		1.07 (0.85–1.35)		1.22 (1.00–1.49)		1.07 (0.85–1.36)	
Q4 (men > 188; women > 174)	745	1.34 (1.09–1.64)	0.015	1.14 (0.89–1.46)	0.610	1.22 (1.00–1.50)	0.053	0.97 (0.76–1.25)	0.745
^8^Baseline dietary fat intake, % E, mean (SD)									
Q1 (< 35)	747	1.00 (ref)	–	1.00 (ref)	–	1.00 (ref)	–	1.00 (ref)	–
Q2 (35–39)	746	1.13 (0.93–1.39)		1.21 (0.96–1.53)		1.05 (0.86–1.29)		1.10 (0.87–1.38)	
Q3 (40–43)	746	1.08 (0.88–1.32)		1.14 (0.90–1.44)		0.95 (0.77–1.16)		0.98 (0.78–1.24)	
Q4 (> 43)	746	1.08 (0.88–1.32)	0.573	1.28 (1.00–1.64)	0.082	0.93 (0.76–1.13)	0.311	1.09 (0.85–1.40)	0.665
^8^Fiber intake, g/day									
Q1 (men < 20; women < 21)	747	1.00 (ref)	–	1.00 (ref)	–	1.00 (ref)	–	1.00 (ref)	–
Q2 (men 20–24; women 21–25)	746	0.87 (0.71–1.06)		1.09 (0.83–1.43)		0.98 (0.80–1.20)		1.30 (0.99–1.71)	
Q3 (men 25–30; women 26–32)	746	0.84 (0.68–1.02)		1.36 (0.97–1.88)		0.87 (0.71–1.06)		1.40 (1.00–1.94)	
Q4 (men > 30 women > 32)	746	0.74 (0.60–0.90)	0.004	**1.62 (1.07–2.46)**	**0.016**	0.75 (0.61–0.92)	0.002	**1.62 (1.07–2.45)**	**0.039**
^8,11^Carbohydrate Quality Index									
Q1 (low)	981	1.00 (ref)	–	1.00 (ref)	–	1.00 (ref)	–	1.00 (ref)	–
Q2	803	0.82 (0.68–0.99)		0.96 (0.77–1.21)		0.81 (0.67–0.97)		0.96 (0.77–1.21)	
Q3	682	0.70 (0.58–0.85)		0.96 (0.74–1.26)		0.67 (0.55–0.82)		0.94 (0.72–1.23)	
Q4 (high)	519	0.61 (0.49–1.75)	< 0.001	1.00 (0.72–1.39)	0.911	0.55 (0.44–0.68)	< 0.001	0.96 (0.69–1.34)	0.713

Bold font is used for significant results for multivariate analysis

*CI* confidence intervals, *CVD*, cardiovascular disease, *DBP* diastolic blood pressure, *E* energy, *MedDiet* Mediterranean diet, *OR* odds ratio, *MET* metabolic equivalent, *Q* quartile, *RAPA* rapid assessment of physical activity, *SBP* systolic blood pressure

For continuous variables, missing data were imputed using simple imputation. For categorical variables, missing data were not imputed and grouped as additional categorical group

^1^Adherence to Mediterranean diet was evaluated using a 17-point scale of adherence to an energy-reduced MedDiet questionnaire (1 point for each item). Participants with an increase of ≥ 5 points from baseline to follow-up were classified in the “*adherent group*”. Participants with ≥ 13 points at baseline and positive increase (≥ 1 point) from baseline to follow-up were additionally classified in the “*adherent group*”. Detailed information is provided in Additional File 1: Figure s1

^2^ORs < 1 was referred as poorer adherence and ORs > 1 was referred as better adherence

^3^Crude model implied bivariate logistic regression

^4^Multivariable model implied multivariable-adjusted logistic regression, adjusted for all characteristics displayed in Table 2 with the addition of the 17-item energy-reduced MedDiet score (data available on Additional File 1: Figure s1)

^5^Family history of premature CVD was defined as any immediate family member deceased younger than 55 years for men and 65 years for women

^6^Number of chronic conditions was calculated by summing the following chronic conditions (1 point for each condition): hypertension, obesity, type 2 diabetes, hypercholesterolemia, cancer, and depression)

^7^Self-reported measure of nervousness and/or aggressiveness behavior was reported on a scale from 1 (very low self-perception) to 10 (very high self-perception)

^8^*P* values for trend were calculated by assigning the median value to each category and treating the resulting variable as continuous

^9^Recruitment year was referred to the period (years) in which participants were recruited, from the date of the first recruited participant (9/05/2013) to the date of the last recruited participant (10/31/2016)

^10^Total workload of center was measured as the number of participants per center

^11^Carbohydrate Quality Index was referred to the quality of dietary carbohydrate intake and was constructed upon four carbohydrate quality domains: total dietary fiber intake (g/day), glycemic index, ratio of carbohydrates from whole grains to carbohydrates from total grains (whole grains + refined grains or their products), and ratio of carbohydrates from solid foods to total carbohydrates (solid carbohydrates + liquid carbohydrates). Quartiles of carbohydrate Quality Index (score) were: Q1: < 9; Q2: 9–10; Q3: 11–12; Q4: > 12

**Table 3:** Association between each individual chronic condition (hypertension, obesity, type 2 diabetes, hypercholesterolemia, cancer, and depression) and good adherence^1^ (increasing ≥ 5 points if baseline < 13 or any increase if baseline ≥ 13) to the MedDiet at 6 and 12 months in the active intervention group of the PREDIMED-Plus trial (*n* = 2,985)

Baseline characteristics	*n*	OR (95% CI) for adherence (increasing ≥ 5 points if baseline < 13p or any increase if baseline ≥ 13p)^1^ to the MedDiet intervention (adherent vs. non-adherent)^2^							
		6 month-follow-up				12 month-follow-up			
		Crude^3^	*p* value	Multivariable^4^	*p* value	Crude^3^	*p* value	Multivariable^4^	*p* value
Health-related characteristics									
Hypertension									
No	488	1.00 (ref)	–	1.00 (ref)	–	1.00 (ref)	–	1.00 (ref)	–
Yes	2497	0.89 (0.74–1.09)	0.260	0.81 (0.65–1.01)	0.063	1.03 (0.84–1.24)	0.802	0.95 (0.76–1.19)	0.655
Obesity									
No	815	1.00 (ref)		1.00 (ref)	–	1.00 (ref)	–	1.00 (ref)	–
Yes	2170	1.00 (0.85–1.18)	0.965	0.81 (0.65–1.01)	0.067	1.08 (0.92–1.27)	0.334	1.10 (0.87–1.37)	0.425
Type 2 diabetes									
No	2154	1.00 (ref)		1.00 (ref)	–	1.00 (ref)	–	1.00 (ref)	–
Yes	831	0.65 (0.55–0.76)	< 0.001	**0.69 (0.55–0.86)**	**0.001**	0.72 (0.61–0.85)	< 0.001	0.82 (0.66–1.02)	0.080
Hypercholesterolemia									
No	897	1.00 (ref)	–	1.00 (ref)	–	1.00 (ref)	–	1.00 (ref)	–
Yes	2088	0.89 (0.76–1.04)	0.154	0.91 (0.76–1.08)	0.288	0.99 (0.84–1.15)	0.853	1.01 (0.85–1.21)	0.876
Cancer									
No	2765	1.00 (ref)	–	1.00 (ref)	–	1.00 (ref)	–	1.00 (ref)	–
Yes	220	0.93 (0.70–1.22)	0.582	0.96 (0.70–1.31)	0.791	0.98 (0.74–1.29)	0.867	1.01 (0.74–1.39)	0.932
Depression									
No	2408	1.00 (ref)	–	1.00 (ref)	–	1.00 (ref)	–	1.00 (ref)	–
Yes	577	0.80 (0.67–0.96)	0.017	**0.80 (0.64–0.99)**	**0.036**	0.73 (0.61–0.87)	0.001	**0.71 (0.57–0.88)**	**0.002**

Bold font is used for significant results for multivariate analysis

*CI* confidence intervals, *MedDiet* Mediterranean diet, *OR* odds ratios

^1^Adherence to Mediterranean diet was evaluated using a 17-point scale of adherence to an energy-reduced MedDiet questionnaire (1 point for each item). Participants with an increase of ≥ 5 points from baseline to follow-up were classified in the “*adherent group*”. Participants with ≥ 13 points at baseline and positive increase (≥ 1 point) from baseline to follow-up were additionally classified in the “*adherent group*”. Detailed information is provided in Additional File 1: Figure s1

^2^ORs < 1 was referred as poorer adherence and ORs > 1 was referred as better adherence

^3^Multivariable model implied multivariable-adjusted logistic regression, adjusted for the same predictors than the logistic model of Table 2 with the addition of hypertension, obesity, type 2 diabetes, hypercholesterolemia, cancer, and depression, and the exclusion of chronic conditions

### Factors of dietary change (12 months)

The factors associated with compliance after 12 months of follow-up identified in the logistic regression models are displayed in Table 2. Greater levels of nervousness and/or aggressiveness, regular and active levels of physical activity, high self-reported self-efficacy to modify diet, and elevated fiber intake at baseline were associated with *better* compliance. In turn, older age (≥ 65 years), being working (vs. retired), unemployed or unable to work (vs. retired), having more than three chronic conditions, and being a current smoker (vs. never smoker) were associated with *poorer* compliance. For study design features, the odds of attaining compliance were significantly *higher* for participants recruited in the second and third years of the trial and participants belonging to field centers with a lower workload. Consistently with the results at 6-month follow-up, participants with depression less likely to comply with the intervention (Table 3). As expected because of a potential ceiling effect, participants with a higher baseline adherence to the erMedDiet showed a smaller dietary change (Additional File 1: Table s2). The factors independently associated with dietary compliance at 12-month follow-up are shown in Fig. 2.

### Sensitivity analyses

We re-ran the models under different assumptions (Additional File 1: Tables s3, s4, and s5). The baseline potential factors which were consistently independently associated with *successful compliance* after 6 and 12 months of follow-up in all sensitivity analyses included high self-reported self-efficacy for diet modification at baseline and higher fiber intake. Consistent potential factors of *poor* compliance included the presence of chronic conditions (both dichotomized and categorized), depression, and higher baseline adherence to erMedDiet scale. High field center workload was the only design feature associated with *poor* adherence. This finding might be related to a suboptimal proportion of staff with respect to participants in the centers with a higher number of participants. Of note, the association between higher fiber intake and higher compliance became inverse when we no longer adjusted for baseline adherence to the 17-item erMedDiet score. This circumstance was also observed when conducting the main analysis.

## Discussion

The PREDIMED-Plus is an intensive nutritional intervention based on major long-term dietary behavioral change aimed to improve participants’ health outcomes, including their risk of cardiovascular events, which also includes regular physical activity and weight-loss goals [31]. We longitudinally examined baseline characteristics related to the attainment of successful dietary behavior changes. The most consistent factors of successful compliance were high baseline perceived self-efficacy to modify diet and high baseline fiber intake. In contrast, the presence of depression and multiple chronic diseases were factors independently associated with poorer compliance.

### Socio-demographics

Previous studies have reported inconclusive results regarding the association between adherence to the MedDiet and socio-demographic characteristics, including sex [9–11, 13, 15, 32–34], or working status [9, 10, 34, 35]. However, married individuals compared to single people seem to respond better to intended dietary changes in previous studies [11, 34], probably because the greater predominance of structured and routine dietary habits among married persons. In our study, we did not find any consistent pattern.

### Health-related characteristics

Individuals with multiple chronic conditions may benefit the most from adhering to a healthy dietary pattern. For instance, the MedDiet has demonstrated numerous positive effects on preventing chronic diseases and improving health outcomes, including type 2 diabetes and depression [8, 36]. Nevertheless, in our study, we found that participants with diabetes and those with a higher number of chronic conditions were less likely to attain high adherence. Prior findings in other studies showed similar results. For example, participants with obesity have been reported to show poorer MedDiet adherence [12], whereas having diabetes and suffering from a greater number of chronic conditions were independent factors of lower compliance [9, 10]. Additionally, our results suggested that a diagnosis of depression at baseline is a strong barrier to modify dietary behavior. Similar findings of poor behavior change among participants with depression have been observed in long-term dietary interventions [37], dietary weight-loss trials [38], and prevention programs for individuals with metabolic syndrome [39]. Moreover, depression has been associated with poorer attendance and early drop-outs in behavioral trials [40]. Potential explanations for these findings may rely on the inherent psychological characteristics of individuals with depression and on the established unhealthy dietary habits that may lead individuals with depression and other chronic conditions to their current health status; nutrition myths or misconceptions related to their diseases [41], and excessive nutritional information received from health care professionals and other sources such as family, friends, or websites [42], may additionally explain these findings. Based on these associations, future dietary behavioral RCTs should carefully collect information about participants’ psychological attributes at baseline, as this information is frequently sub-optimally collected [43]. Exclusion of individuals with depression would allow to identify probable candidates for early drop-outs and low compliance, and ensure a significant contrast between the intervention and control arms of future trials. On the other hand, careful design of RCTs specifically targeting these participants with depression may be a desirable approach so that they could also beneficiate from tailored dietary interventions, but they will need very specific and particularly intensive intervention protocols. A more intense and specific dietary counseling with adapted information and personalized messages for individuals with chronic conditions is also highly warranted.

### Lifestyle and behavior

In our study, high self-efficacy, a social cognitive theory component, was an important predictor of better compliance to the erMedDiet. According to Bandura et al. [44], an individual’s beliefs according to his/her abilities determine certain behaviors. In this case, participants’ beliefs regarding personal success on the desired outcome (dietary changes) may determine their effort level. Hence, participants with high perceived self-efficacy are expected to pursue dietary modifications until successful dietary change is achieved. Consistent with our findings, high self-efficacy has been a promising predictor in weight-loss interventions and physical activity among overweight/obese populations [45]. Moreover, long-term maintenance of high self-efficacy has been associated with greater weight loss [46]. This evidence emphasizes the importance of collecting this information at baseline and incorporating specific strategies to maintain a high self-efficacy level throughout the follow-up period of the interventions. Negotiated goal setting, continuous persuasion, permanent performance feedback, shared decision-making, alternatives to overcome struggles faced by participants to improve their diets, and problem-solving strategies are different approaches which may improve and maintain self-efficacy along the trial. These strategies have been key in previous RCTs, such as the PREDIMED, to successfully improve participants’ adherence to the MedDiet in the intervention groups [8].

### Dietary characteristics

Participants with a poorer baseline adherence to the erMedDiet score, had greater room for improvement whereas participants with higher adherence at baseline may face a ceiling effect. Therefore, it is not surprising to find better achievements among those with poorer scores at baseline. Interestingly, we found that higher fiber intake was a factor independently associated with better dietary changes. In previous studies, fiber intake resulted a robust predictor of weight loss and beneficial food-related behavioral changes [47, 48]. Participants with high fiber intake at baseline may be more health conscious and they may be more likely to better adapt to fiber-rich food patterns such, as the Mediterranean diet than individuals with poorer baseline fiber intake. This finding was observed after adjustment for the baseline adherence to the 17-item erMedDiet score. This is important, given that participants with poorer adherence to the MedDiet usually tend to have lower fiber intake, as it was the case in our study.

The current study has some limitations. First, information about the participants’ diet and health conditions was self-reported, and recall bias and misreporting may be present when using self-reported information. Nevertheless, their self-reported changes were paralleled by objective changes in cardiovascular risk factors as reported elsewhere [17, 30]. Second, the observational nature of the study limits causal inferences. Third, although we tested several characteristics to predict behavior change and adjusted for a wide array of potential confounding factors, failure to control for other factors may be possible and we cannot exclude residual confounding. Nevertheless, we examined multiple potential factors chosen according to the existing literature and some of our previous studies. And fourth, the PREDIMED-Plus includes an overweight/obese community-dwelling population with metabolic syndrome which is not representative of the general population. However, this population is becoming more predominant in developed and developing countries, increasing the actual practical interest of our findings. Despite the aforementioned limitations, the strength of our study relies on the evaluation of a high number of baseline candidate factors, the inclusion of several sensitivity analyses that corroborated our findings, and the use of high-quality prospective data with a very high retention rate from one of the largest nutritional trials, the PREDIMED-Plus trial.

## Conclusions

The present study provides a better understanding of factors associated with successful compliance to a dietary intervention. Recruitment of individuals highly motivated to change their diet and of those who follow a fiber-rich dietary pattern but even so, they poorly adhere at baseline to the intended diet would potentially increase the needed contrast between the arms of a dietary intervention trial. Participants with multiple chronic conditions, particularly depression, should receive tailored protocols and specific attention, because they are not likely to respond to conventional interventions. Future studies should investigate strategies to promote better compliance among those individuals with features which predict poor compliance to the intended dietary behavior changes.

## Supplementary Information

Below is the link to the electronic supplementary material.

Supplementary file1 (DOCX 202 KB)

## Data Availability

There are restrictions on the availability of data for the PREDIMED-Plus trial, due to the signed consent agreements around data sharing, which only allow access to external researchers for studies following the project purposes. Requestors wishing to access the PREDIMED-Plus trial data used in this study can make a request to the PREDIMED-Plus trial Steering Committee chair: predimed_plus_scommitte@googlegroups.com. The request will then be passed to members of the PREDIMED-Plus Steering Committee for deliberation.

## References

[1] Pan A, Lin X, Hemler E, Hu FB (2018). Diet and cardiovascular disease: advances and challenges in population-based studies. Cell Metab.

[2] Satija A, Stampfer MJ, Rimm EB (2018). Perspective: are large, simple trials the solution for nutrition research?. Adv Nutr.

[3] Howard BV, Van Horn L, Hsia J (2006). Low-fat dietary pattern and risk of cardiovascular disease: the Women’s Health Initiative randomized controlled dietary modification trial. JAMA.

[4] Prentice RL, Caan B, Chlebowski RT (2006). Low-fat dietary pattern and risk of invasive breast cancer: the Women’s Health Initiative randomized controlled dietary modification trial. JAMA.

[5] Orloff JN, Aronne LJ, Shukla AP (2018). The challenge of meeting prescribed carbohydrate intake goals in low-carbohydrate diet studies. Am J Clin Nutr.

[6] Kjelsberg MO (1982). Multiple risk factor intervention trial: risk factor changes and mortality results. JAMA.

[7] Willett WC, Stampfer MJ (1990). Dietary fat and cancer: another view. Cancer Causes Control.

[8] Fernández-Lázaro CI, Ruiz-Canela M, Martínez-González MÁ (2021). Deep dive to the secrets of the PREDIMED trial. Curr Opin Lipidol.

[9] Zazpe I, Estruch R, Toledo E (2010). Predictors of adherence to a Mediterranean-type diet in the PREDIMED trial. Eur J Nutr.

[10] Downer MK, Gea A, Stampfer M (2016). Predictors of short- and long-term adherence with a Mediterranean-type diet intervention: the PREDIMED randomized trial. Int J Behav Nutr Phys Act.

[11] Hu EA, Toledo E, Diez-Espino J (2013). Lifestyles and risk factors associated with adherence to the mediterranean diet: a baseline assessment of the PREDIMED trial. PLoS ONE.

[12] Patino-Alonso MC, Recio-Rodríguez JI, Belio JFM (2014). Factors associated with adherence to the Mediterranean diet in the adult population. J Acad Nutr Diet.

[13] Ruggiero E, Di Castelnuovo A, Costanzo S (2019). Socioeconomic and psychosocial determinants of adherence to the Mediterranean diet in a general adult Italian population. Eur J Public Health.

[14] Peng W, Goldsmith R, Berry EM (2016). Demographic and lifestyle factors associated with adherence to the Mediterranean diet in relation to overweight/obesity among Israeli adolescents: findings from the Mabat Israeli national youth health and nutrition survey. Public Health Nutr.

[15] Grosso G, Marventano S, Buscemi S (2013). Factors associated with adherence to the Mediterranean diet among adolescents living in Sicily, southern Italy. Nutrients.

[16] Martínez-González MA, Buil-Cosiales P, Corella D (2019). Cohort Profile: Design and methods of the PREDIMED-Plus randomized trial. Int J Epidemiol.

[17] Sayón-Orea C, Razquin C, Bulló M (2019). Effect of a Nutritional and Behavioral Intervention on energy-reduced mediterranean diet adherence among patients with metabolic syndrome: interim analysis of the PREDIMED-plus randomized clinical trial. JAMA.

[18] Schröder H, Cárdenas-Fuentes G, Martínez-González MA (2018). Effectiveness of the physical activity intervention program in the PREDIMED-Plus study: a randomized controlled trial. Int J Behav Nutr Phys Act.

[19] World Health Organization (2021) Physical activity and older adults. https://www.who.int/ncds/prevention/physical-activity/factsheet_olderadults/en. Accessed 1 Mar 2020

[20] Alberti KGMM, Eckel RH, Grundy SM (2009). Harmonizing the metabolic syndrome: a joint interim statement of the international diabetes federation task force on epidemiology and prevention; National heart, lung, and blood institute; American heart association; World heart federation; International. Circulation.

[21] Schröder H, Zomeño MD, Martínez-González MA (2021). Validity of the energy-restricted Mediterranean Diet Adherence Screener. Clin Nutr.

[22] de la Fuente-Arrillaga C, Vázquez Ruiz Z, Bes-Rastrollo M (2010). Reproducibility of an FFQ validated in Spain. Public Health Nutr.

[23] Fernández-Ballart JD, Piñol JL, Zazpe I (2010). Relative validity of a semi-quantitative food-frequency questionnaire in an elderly Mediterranean population of Spain. Br J Nutr.

[24] Moreiras O, Carbajal A, Cabrera L, Cuadrado C (2011). Tablas de Composición de Alimentos (Food Composition Tables).

[25] Mataix-Verdú J, García-Diaz L, Manas M (2003). Tabla de Composición de Alimentos (Spanish Food Composition Tables).

[26] Topolski TD, LoGerfo J, Patrick DL (2006). The rapid assessment of physical activity (RAPA) among older adults. Prev Chronic Dis.

[27] Molina L, Sarmiento M, Peñafiel J (2017). Validation of the regicor short physical activity questionnaire for the adult population. PLoS ONE.

[28] Martínez-González MA, López-Fontana C, Varo JJ (2005). Validation of the Spanish version of the physical activity questionnaire used in the Nurses’ Health Study and the Health Professionals’ Follow-up Study. Public Health Nutr.

[29] Willet WC (2013). Nutritional epidemiology.

[30] Martínez-González MA, Fernandez-Lazaro CI, Toledo E (2019). Carbohydrate quality changes and concurrent changes in cardiovascular risk factors: a longitudinal analysis in the PREDIMED-plus randomized trial. Am J Clin Nutr.

[31] Martínez-González MÁ, Corella D, Salas-Salvadó J (2012). Cohort profile: design and methods of the PREDIMED study. Int J Epidemiol.

[32] Sánchez-Villegas A, Martínez JA, De Irala J, Martínez-González MA (2002). Determinants of the adherence to an “a priori” defined Mediterranean dietary pattern. Eur J Nutr.

[33] González CA, Argilaga S, Agudo A (2002). Diferencias sociodemográficas en la adhesión al patrón de dieta mediterránea en poblaciones de España (sociodemographic differences in adherence to the Mediterranean dietary pattern in Spanish populations). Gac Sanit.

[34] Jurado D, Burgos-Garrido E, Diaz FJ (2012). Adherence to the Mediterranean dietary pattern and personality in patients attending a primary health center. J Acad Nutr Diet.

[35] Marventano S, Godos J, Platania A (2018). Mediterranean diet adherence in the Mediterranean healthy eating, aging and lifestyle (MEAL) study cohort. Int J Food Sci Nutr.

[36] Sánchez-Villegas A, Delgado-Rodríguez M, Alonso A (2009). Association of the Mediterranean dietary pattern with the incidence of depression: the Seguimiento Universidad de Navarra/University of Navarra follow-up (SUN) Cohort. Arch Gen Psychiatry.

[37] Wang JB, Pierce JP, Ayala GX (2015). Baseline depressive symptoms, completion of study assessments, and behavior change in a long-term dietary intervention among breast cancer survivors. Ann Behav Med.

[38] Somerset SM, Graham L, Markwell K (2011). Depression scores predict adherence in a dietary weight loss intervention trial. Clin Nutr.

[39] Susin N, De Melo BR, Ludwig MWB (2016). Predictors of adherence in a prevention program for patients with metabolic syndrome. J Health Psychol.

[40] Moroshko I, Brennan L, O’Brien P (2011). Predictors of dropout in weight loss interventions: a systematic review of the literature. Obes Rev.

[41] Lesser LI, Mazza MC, Lucan SC (2015). Nutrition myths and healthy dietary advice in clinical practice. Am Fam Physician.

[42] Ball L, Davmor R, Leveritt M (2016). Understanding the nutrition care needs of patients newly diagnosed with type 2 diabetes: a need for open communication and patient-focussed consultations. Aust J Prim Health.

[43] Somerset SM, Markwell K, Al-Foraih M (2013). A systematic review of baseline psychosocial characterisation in dietary randomised controlled trials for weight loss. Eur J Clin Nutr.

[44] Bandura A (1997) Self-efficacy: the exercise of control. W.H. Freeman and Company, New York

[45] Teixeira PJ, Carraça EV, Marques MM (2015). Successful behavior change in obesity interventions in adults: a systematic review of self-regulation mediators. BMC Med.

[46] Warziski MT, Sereika SM, Styn MA (2008). Changes in self-efficacy and dietary adherence: the impact on weight loss in the PREFER study. J Behav Med.

[47] Miketinas DC, Bray GA, Beyl RA (2019). Fiber intake predicts weight loss and dietary adherence in adults consuming calorie-restricted diets: the POUNDS lost (Preventing Overweight Using Novel Dietary Strategies) study. J Nutr.

[48] Hiel S, Bindels LB, Pachikian BD (2019). Effects of a diet based on inulin-rich vegetables on gut health and nutritional behavior in healthy humans. Am J Clin Nutr.

