# Supplementary material for: Factors associated with successful dietary changes in an energy-reduced Mediterranean diet intervention: a longitudinal analysis in the PREDIMED-Plus trial
Source: Eur J Nutr. 2021 Nov 30;61(3):1457–75. doi: 10.1007/s00394-021-02697-8 (PMC8921156; doi:10.1007/s00394-021-02697-8)
Supplement: Supplementary file 1 — Supplementary file1 (DOCX 202 KB) [file 394_2021_2697_MOESM1_ESM.docx]

**SUPPLEMENTARY INFORMATION**

**Factors associated with successful dietary changes in an energy-reduced Mediterranean diet intervention: A longitudinal analysis in the PREDIMED-PLUS trial**

Cesar I Fernandez-Lazaro, Estefanía Toledo, Pilar Buil-Cosiales, Jordi Salas-Salvadó, Dolores Corella, Montserrat Fitó, J, et al.

**Table s1.** Description of the 17-item energy-reduced Mediterranean Diet questionnaire.

**Table s2**. Association between the 17-point scale of adherence to MedDiet and attaining good adherence^1^ (increasing ≥5 points if baseline<13 or any increase if baseline ≥13) to the MedDiet intervention at 6 and 12 months in the active intervention group of the PREDIMED-PLUS trial (n=2,985). Odds Ratios (OR) and 95% confidence intervals (95% CI).

**Table s3.** Analyses only among participants without missing values at baseline in the active intervention group of the PREDIMED-PLUS trial (n=2,796). Participants with any missing value were excluded (n=189). Odds Ratios (OR) and 95% confidence intervals (95% CI) of attaining good adherence^1^ (increasing ≥5 points if baseline <13 or any increase if baseline ≥13) to the MedDiet intervention at 6 and 12 months of follow-up.

**Table s4.** Analyses only among participants with baseline score <13 points in the active intervention group of the PREDIMED-PLUS trial (n=2,793). Participants with baseline score ≥13 points at baseline were excluded (n=192). Odds Ratios (OR) and 95% confidence intervals (95% CI) of attaining good adherence^1^ (increasing the score in ≥5 points from baseline) to the MedDiet intervention at 6 and 12 months of follow-up.

**Table s5.** Analyses using an alternate definition of good adherence^1^: score >12 points to the MedDiet intervention. Odds Ratios (OR) and 95% confidence intervals (95% CI) of attaining good adherence^1^ (score >12 points) to the MedDiet intervention at 6 and 12 months of follow-up in the active intervention group of the PREDIMED-PLUS trial^1^ (n=2,985).

**Figure s1.** Outcome definition of adherent and non-adherent participants on the active group of the PREDIMED-PLUS trial.

**Figure s2.** Flow chart of the participants of thw study. The PREDIMED-Plus trial.

**TABLES**

| **Table s1.** Description of the 17-item energy-reduced Mediterranean Diet questionnaire. | | |
| --- | --- | --- |
|  | **Item** | **Compliance with the item** |
| *1* | Extra-virgin olive oil for cooking | Use only extra-virgin olive oil for cooking, salad dressings, and spreads. |
| *2* | Vegetables | Consume ≥2 portions (200g) of vegetables per day, at least one of them raw. |
| *3* | Fruits | Consume ≥3 portions of fruit per day (including natural fruit juices). |
| *4* | Red and processed meat | Consume ≤1 serving (100-150g) of red meat, hamburgers, or meat products (ham, sausage, etc.) per week. |
| *5* | Butter, margarine, cream | Consume less than 1 serving (12g) of butter, margarine or cream per week. |
| *6* | Sugar sweetened beverages | Consume less than one sugary beverage or sugar-sweetened fruit juice per week. |
| *7* | Legumes | Consume ≥3 servings (150g) of legumes per week. |
| *8* | Fish and seafood | Consume ≥3 servings of fish (100-150g) or shellfish (200g) per week. |
| *9* | Sweets and pastries | Consume <3 non-homemade sweets or pastries, such as cakes, cookies, sponge cake, or custard, per week |
| *10* | Nuts | Consume ≥3 (30g) servings of nuts (including peanuts) per week. |
| *11* | Preference white over red meat | Consume chicken, turkey or rabbit meat instead of beef, pork, hamburgers or sausages. |
| *12* | Sofrito | Use sofrito ≥2 times per week (Sofrito: tomato and onion sauce, with garlic and aromatic herbs, simmered in olive oil.). |
| *13* | Avoidance of adding sugar to beverages | Do not add sugar to beverages . |
| *14* | White bread | Reduce consumption of white bread to ≤1 serving (75g)/day |
| *15* | Whole grains | Consume whole grain cereals and whole grain pasta ≥5 times per week. |
| *16* | Refined cereals | Reduce consumption of non-whole grain pasta or rice <3 servings per week. |
| *17* | Wine | Consume 2-3 glasses (100ml/glass) of wine per day (men) or 1-2 glasses of wine per day (women). |

| **Table s2**. Association between the 17-point scale of adherence to MedDiet and attaining good adherence^1^ (increasing ≥5 points if baseline<13 or any increase if baseline ≥13) to the MedDiet intervention at 6 and 12 months in the active intervention group of the PREDIMED-PLUS trial (n=2,985). Odds Ratios (OR) and 95% confidence intervals (95% CI). | | | | | | | | | | | | |
| --- | --- | --- | --- | --- | --- | --- | --- | --- | --- | --- | --- | --- |
|  |  |  | **OR (95 % CI) for adherence (increasing ≥5 points if baseline <13p or any increase if baseline ≥13p)^1^  to the MedDiet intervention (adherent vs. non-adherent) ^2^** | | | | | | | | | |
|  |  | **6 month-follow-up** | | | | |  | **12 month-follow-up** | | | |  |
| Baseline characteristics | **n** | **Crude^3^** | | ***p*-value** | **Multivariable^4^** | ***p*-value** |  | **Crude^3^** | ***p*-value** | **Multivariable^4^** | ***p*-value** |  |
| **Dietary pattern** |  |  | |  |  |  |  |  |  |  |  |  |
| ^8^17-item energy-reduced MedDiet score |  |  | |  |  |  |  |  |  |  |  |  |
| Q1 (<8) | 1135 | 1.00 (ref) | | -- | 1.00 (ref) | -- |  | 1.00 (ref) | -- | 1.00 (ref) | -- |  |
| Q2 (8) | 416 | 0.42 (0.33-0.53) | |  | **0.39 (0.30-0.50)** |  |  | 0.43 (0.34-0.55) |  | **0.41 (0.32-0.52)** |  |  |
| Q3 (9-10) | 744 | 0.23 (0.19-0.28) | |  | **0.20 (0.16-0.24)** |  |  | 0.25 (0.21-0.31) |  | **0.21 (0.17-0.26)** |  |  |
| Q4 (>10) | 690 | 0.13 (0.10-0.16) | | < 0.001 | **0.10 (0.08-0.13)** | **< 0.001** |  | 0.12 (0.10-0.15) | < 0.001 | **0.09 (0.07-0.11)** | **< 0.001** |  |

Abbreviations: MedDiet, Mediterranean diet

^1^Adherence to Mediterranean diet was evaluated using a 17-point scale of adherence to an energy-reduced MedDiet questionnaire (1 point for each item). Participants with an increase of ≥5 points from baseline to follow-up were classified in the “*adherent group*”. Participants with ≥13 points at baseline and positive increase (≥1 point) from baseline to follow-up were additionally classified in the “*adherent group*”. Detailed information is provided in Supplemental Material Figure 1.

^2^ORs <1 was referred as poorer adherence and ORs >1 was referred as better adherence.

^3^Crude model implied bivariate logistic regression.

^4^Multivariable model implied multivariable-adjusted logistic regression, adjusted for the rest of the characteristics displayed in Table 2.

| **Table s3.** **Analyses only among participants without missing values at baseline in the active intervention group of the PREDIMED-PLUS trial (n=2,796). Participants with any missing value were excluded (n=189).** Odds Ratios (OR) and 95% confidence intervals (95% CI) of attaining good adherence^1^ (increasing ≥5 points if baseline <13 or any increase if baseline ≥13) to the MedDiet intervention at 6 and 12 months of follow-up. | | | | | | | |  |
| --- | --- | --- | --- | --- | --- | --- | --- | --- |
|  |  | | **OR (95 % CI) for adherence (increasing ≥5 points if baseline <13p or any increase if baseline ≥13p)^1^ to the MedDiet intervention  (adherent vs. non-adherent) ^2^** | | | | |  |
|  |  | | **6-month follow-up** | |  | **12-month follow-up** | |  |
| Baseline characteristics | **n** | | **Multivariable^3^** | ***p*-value** |  | **Multivariable^3^** | ***p*-value** |  |
| **Socio-demographics** |  | |  |  |  |  |  |  |
| Sex |  | |  |  |  |  |  |  |
| Men | 1445 | | 1.00 (ref) | -- |  | 1.00 (ref) | -- |  |
| Women | 1351 | | 1.24 (0.96 - 1.60) | 0.100 |  | 1.09 (0.85 - 1.41) | 0.498 |  |
| Age, years |  | |  |  |  |  |  |  |
| < 65 | 1322 | | 1.00 (ref) | -- |  | 1.00 (ref) | -- |  |
| ≥ 65 | 1474 | | 0.85 (0.68 - 1.06) | 0.148 |  | **0.79 (0.63 - 0.98)** | **0.034** |  |
| Marital status |  | |  |  |  |  |  |  |
| Married | 2125 | | 1.00 (ref) | -- |  | 1.00 (ref) | -- |  |
| Single | 151 | | **0.66 (0.45 - 0.97)** | **0.034** |  | 0.83 (0.56 - 1.22) | 0.341 |  |
| Widowed | 297 | | 0.94 (0.70 - 1.26) | 0.664 |  | 0.90 (0.67 - 1.21) | 0.493 |  |
| Others | 223 | | 0.89 (0.64 - 1.22) | 0.468 |  | 0.77 (0.56 - 1.06) | 0.111 |  |
| Attained education level |  | |  |  |  |  |  |  |
| College/university | 604 | | 1.00 (ref) | -- |  | 1.00 (ref) |  |  |
| Secondary | 856 | | 0.91 (0.72 - 1.16) | 0.442 |  | 0.82 (0.64 - 1.04) | 0.100 |  |
| Primary or less | 1336 | | 1.04 (0.82 - 1.32) | 0.752 |  | 0.99 (0.78 - 1.26) | 0.949 |  |
| Occupation |  | |  |  |  |  |  |  |
| Retired | 1562 | | 1.00 (ref) | -- |  | 1.00 (ref) | -- |  |
| Working | 602 | | 0.92 (0.70 - 1.20) | 0.526 |  | **0.73 (0.56 - 0.96)** | **0.022** |  |
| Unemployed or unable to work | 229 | | 0.80 (0.57 - 1.13) | 0.204 |  | 0.71 (0.51 - 1.01) | 0.057 |  |
| Housewife | 403 | | 0.82 (0.62 - 1.09) | 0.173 |  | 0.89 (0.67 - 1.18) | 0.415 |  |
| Number of people in household | 2796 | | 0.96 (0.88 - 1.05) | 0.387 |  | 0.99 (0.91 - 1.09) | 0.897 |  |
| **Health-related characteristics** |  | |  |  |  |  |  |  |
| ^4^Family history of premature CVD |  | |  |  |  |  |  |  |
| No | 2442 | | 1.00 (ref) | -- |  | 1.00 (ref) | -- |  |
| Yes | 354 | | 1.04 (0.81 - 1.33) | 0.770 |  | 0.94 (0.73 - 1.21) | 0.644 |  |
| ^5^Number of chronic conditions |  | |  |  |  |  |  |  |
| ≤ 3 | 2124 | | 1.00 (ref) | -- |  | 1.00 (ref) | -- |  |
| > 3 | 672 | | **0.64 (0.52 - 0.79)** | **< 0.001** |  | **0.77 (0.62 - 0.95)** | **0.014** |  |
| ^6,7^Self-reported measure of nervousness and/or aggressiveness behavior, score |  | |  |  |  |  |  |  |
| Q1(<4) | 753 | | 1.00 (ref) | -- |  | 1.00 (ref) | -- |  |
| Q2 (4-5) | 1014 | | 0.90 (0.72 - 1.11) |  |  | 1.04 (0.84 - 1.28) |  |  |
| Q3 (6) | 336 | | 0.87 (0.65 - 1.16) |  |  | 1.06 (0.79 - 1.42) |  |  |
| Q4 (>6) | 693 | | 1.14 (0.90 - 1.44) | 0.584 |  | **1.42 (1.12 - 1.80)** | **0.013** |  |
| Body weight, (per 5 kg) | 2796 | | 1.02 (0.96 - 1.09) | 0.481 |  | 1.03 (0.97 - 1.09) | 0.351 |  |
| Waist circumference (per 5 cm) | 2796 | | 1.01 (0.94 - 1.09) | 0.738 |  | 0.98 (0.91 - 1.06) | 0.598 |  |
| SBP (per 5mm Hg) | 2796 | | 1.03 (1.00 - 1.06) | 0.088 |  | 1.01 (0.98 - 1.04) | 0.698 |  |
| DBP (per 5mm Hg) | 2796 | | 1.01 (0.96 - 1.06) | 0.761 |  | 1.01 (0.96 - 1.07) | 0.614 |  |
| Fasting blood glucose (per 10 mg/dL) | 2796 | | 1.01 (0.98 - 1.05) | 0.413 |  | 1.01 (0.97 - 1.04) | 0.719 |  |
| **Study Design Features** |  | |  |  |  |  |  |  |
| ^8^Recruitment year |  | |  |  |  |  |  |  |
| < 1^st^ | 222 | | 1.00 (ref) | -- |  | 1.00 (ref) | -- |  |
| 1^st^-2^nd^ | 736 | | 1.25 (0.89 - 1.76) | 0.204 |  | **1.68 (1.19 - 2.37)** | **0.003** |  |
| 2^nd^-3^rd^ | 1421 | | **1.49 (1.07 - 2.08)** | **0.019** |  | **1.75 (1.25 - 2.44)** | **0.001** |  |
| > 3^rd^ | 417 | | 1.13 (0.77 - 1.66) | 0.520 |  | **1.48 (1.01 - 2.17)** | **0.044** |  |
| ^9^Total workload of center, participants in intervention group |  | |  |  |  |  |  |  |
| Below median (≤119) | 1417 | | 1.00 (ref) | -- |  | 1.00 (ref) | -- |  |
| Above median (>119) | 1379 | | **0.76 (0.64 - 0.90)** | **0.002** |  | **0.82 (0.69 - 0.97)** | **0.022** |  |
| **Lifestyle behavior** |  | |  |  |  |  |  |  |
| Physical activity |  | |  |  |  |  |  |  |
| ^7^METs-min/wk |  | |  |  |  |  |  |  |
| Q1 (<840) | 727 | | 1.00 (ref) | -- |  | 1.00 (ref) | -- |  |
| Q2 (840-1829) | 670 | | **1.35 (1.05 - 1.72)** |  |  | 1.04 (0.81 - 1.33) |  |  |
| Q3 (1830-3356) | 712 | | **1.30 (1.01 - 1.68)** |  |  | 1.11 (0.86 - 1.43) |  |  |
| Q4 (>3356) | 687 | | 1.11 (0.85 - 1.46) | 0.986 |  | 1.17 (0.89 - 1.53) | 0.249 |  |
| RAPA test |  | |  |  |  |  |  |  |
| Level 1 (sedentary or under-active) | 520 | | 1.00 (ref) | -- |  | 1.00 (ref) | -- |  |
| Level 2 (under-active regular – light activities) | 1008 | | 1.00 (0.78 - 1.27) | 0.972 |  | 1.02 (0.80 - 1.31) | 0.844 |  |
| Level 3 (under-active regular) | 500 | | 1.14 (0.85 - 1.53) | 0.394 |  | **1.42 (1.05 - 1.91)** | **0.022** |  |
| Level 4 (active) | 768 | | 1.08 (0.81 - 1.44) | 0.605 |  | **1.42 (1.06 - 1.90)** | **0.017** |  |
| ^7^Chair test 30s, repeats |  | |  |  |  |  |  |  |
| Q1 (<12) | 906 | | 1.00 (ref) | -- |  | 1.00 (ref) | -- |  |
| Q2 (12-13) | 622 | | 1.02 (0.81 - 1.29) |  |  | 0.96 (0.76 - 1.21) |  |  |
| Q3 (14-16) | 699 | | 0.97 (0.77 - 1.22) |  |  | 0.98 (0.77 - 1.24) |  |  |
| Q4 (>16) | 569 | | 0.90 (0.70 - 1.16) | 0.382 |  | 0.85 (0.66 - 1.09) | 0.245 |  |
| Smoking status, n(%) |  | |  |  |  |  |  |  |
| Never smokers | 1257 | | 1.00 (ref) | -- |  | 1.00 (ref) | -- |  |
| Current smokers | 378 | | **0.74 (0.56 - 0.97)** | **0.032** |  | **0.68 (0.51 - 0.90)** | **0.007** |  |
| Former smokers | 1161 | | 0.98 (0.80 - 1.20) | 0.819 |  | 0.93 (0.76 - 1.15) | 0.506 |  |
| Alcohol intake other than wine |  | |  |  |  |  |  |  |
| Abstainers | 1385 | | 1.00 (ref) | -- |  | 1.00 (ref) | -- |  |
| ≤ 5 g/d | 750 | | 0.91 (0.75 - 1.12) | 0.379 |  | 0.95 (0.77 - 1.16) | 0.583 |  |
| > 5 g/d | 661 | | 0.93 (0.72 - 1.20) | 0.577 |  | 1.00 (0.77 - 1.29) | 0.995 |  |
| ^7^Sleeping, hours/d |  | |  |  |  |  |  |  |
| Q1 (<7) | 909 | | 1.00 (ref) | -- |  | 1.00 (ref) | -- |  |
| Q2 (7) | 919 | | 0.96 (0.78 - 1.18) |  |  | 0.90 (0.73 - 1.11) |  |  |
| Q3 (8) | 725 | | 0.85 (0.68 - 1.05) |  |  | 0.99 (0.79 - 1.23) |  |  |
| Q4 (>8) | 243 | | 1.15 (0.83 - 1.58) | 0.723 |  | 0.99 (0.72 - 1.36) | 0.990 |  |
| Self-efficacy for diet modification |  | |  |  |  |  |  |  |
| Little or some | 703 | | 1.00 (ref) | -- |  | 1.00 (ref) | -- |  |
| High | 2093 | | **1.51 (1.24 - 1.84)** | **< 0.001** |  | **1.69 (1.39 - 2.06)** | **< 0.001** |  |
| **Total energy and nutrient intake** | |  |  |  |  |  |  | |
| ^7^Total energy intake, kcal/d |  | |  |  |  |  |  |  |
| Q1 (men <2121; women <1878) | 709 | | 1.00 (ref) | -- |  | 1.00 (ref) | -- |  |
| Q2 (men 2121-2475; women: 1878-2210) | 697 | | 1.00 (0.78 - 1.29) |  |  | 1.04 (0.81 - 1.34) |  |  |
| Q3 (men 2476-2883; women: 2211-2567) | 693 | | 1.02 (0.78 - 1.34) |  |  | 1.11 (0.85 - 1.45) |  |  |
| Q4 (men >2883; women: >2567) | 697 | | 0.81 (0.60 - 1.11) | 0.176 |  | 0.87 (0.64 - 1.19) | 0.394 |  |
| Predefined limits of energy intake (Willet), kcal/d |  | |  |  |  |  |  |  |
| Within limits (men 800-4000; women 500-3500) | 2730 | | 1.00 (ref) |  |  | 1.00 (ref) | -- |  |
| Beyond limits (men < 800 or >4000; women <500 or >3500) | 66 | | 0.67 (0.38 - 1.19) | 0.171 |  | 1.09 (0.61 - 1.95) | 0.783 |  |
| ^7^Fruit + vegetable consumption, g/d |  | |  |  |  |  |  |  |
| Q1 (men, <472; women <543) | 71 | | 1.00 (ref) | -- |  | 1.00 (ref) | -- |  |
| Q2 (men 472-625; women 544-700) | 689 | | **1.35 (1.05 - 1.73)** |  |  | 0.95 (0.74 - 1.22) |  |  |
| Q3 (men 626-794; women 701-887) | 704 | | 0.99 (0.75 - 1.31) |  |  | 0.94 (0.71 - 1.25) |  |  |
| Q4 (men >794; women >887) | 702 | | 0.97 (0.72 - 1.32) | 0.445 |  | 0.84 (0.62 - 1.15) | 0.276 |  |
| ^7^Meat consumption, g/d |  | |  |  |  |  |  |  |
| Q1 (men <114; women <105) | 705 | | 1.00 (ref) | -- |  | 1.00 (ref) | -- |  |
| Q2 (men 114-148; women 105-137) | 689 | | **1.35 (1.07 - 1.72)** |  |  | 1.09 (0.86 - 1.38) |  |  |
| Q3 (men 149-188; women 138-174) | 700 | | 1.05 (0.83 - 1.34) |  |  | 1.02 (0.80 - 1.30) |  |  |
| Q4 (men >188; women >174) | 702 | | 1.18 (0.91 - 1.53) | 0.509 |  | 0.98 (0.76 - 1.27) | 0.784 |  |
| ^7^Baseline Dietary fat intake, % E, mean (SD) |  | |  |  |  |  |  |  |
| Q1 (<35) | 709 | | 1.00 (ref) | -- |  | 1.00 (ref) | -- |  |
| Q2 (35-38) | 699 | | 1.19 (0.94 - 1.51) |  |  | 1.14 (0.90 - 1.45) |  |  |
| Q3 (39-43) | 698 | | 1.12 (0.88 - 1.43) |  |  | 0.98 (0.77 - 1.25) |  |  |
| Q4 (>43) | 690 | | 1.29 (1.00 - 1.67) | 0.080 |  | 1.15 (0.89 - 1.48) | 0.502 |  |
| ^7^Fiber intake, g/d |  | |  |  |  |  |  |  |
| Q1 (men <20; women < 21) | 699 | | 1.00 (ref) | -- |  | 1.00 (ref) | -- |  |
| Q2 (men 20-24; women 21-25) | 701 | | 1.08 (0.82 - 1.43) |  |  | 1.29 (0.97 - 1.71) |  |  |
| Q3 (men 25-30; women 26-32) | 697 | | 1.36 (0.97 - 1.92) |  |  | 1.36 (0.97 - 1.92) |  |  |
| Q4 (men >30 women >32) | 699 | | **1.70 (1.11 - 2.61)** | **0.009** |  | **1.60 (1.05 - 2.46)** | **0.047** |  |
| ^7,10^Carbohydrate Quality Index |  | |  |  |  |  |  |  |
| Q1 (low) | 922 | | 1.00 (ref) | -- |  | 1.00 (ref) | -- |  |
| Q2 | 745 | | 0.92 (0.73 - 1.17) |  |  | 1.01 (0.80 - 1.28) |  |  |
| Q3 | 643 | | 0.92 (0.70 - 1.21) |  |  | 0.95 (0.72 - 1.25) |  |  |
| Q4 (high) | 486 | | 0.97 (0.69 - 1.36) | 0.737 |  | 1.05 (0.75 - 1.48) | 0.914 |  |

Abbreviations: CVD, cardiovascular disease; DBP, diastolic blood pressure; E, energy; M, months; MedDiet, Mediterranean diet; MET, metabolic equivalent; Q, quartile; RAPA, rapid assessment of physical activity; SBP, systolic blood pressure.

^1^Adherence to Mediterranean diet was evaluated using a 17-point scale of adherence to an energy-reduced MedDiet questionnaire (1 point for each item). Participants with an increase of ≥5 points from baseline to follow-up were classified in the “adherent group”. Participants with ≥13 points at baseline and positive increase (≥1 point) from baseline to follow-up were additionally classified in the “adherent group”. Participants with any missing value were exclude from the analyses.

^2^ORs <1 was referred as poorer adherence and ORs >1 was referred as better adherence.

^3^Multivariable model implied multivariable-adjusted logistic regression, adjusted for all characteristics displayed in Supplemental Table 2 with the addition of the 17-item energy-reduced MedDiet score and the exclusion of hypertension, obesity, type 2 diabetes, hypercholesterolemia, cancer, and depression.

^4^Family history of premature CVD was defined as any immediate family member deceased younger than 55 years for men and 65 years for women.

^5^Number of chronic conditions was calculated by summing the following chronic conditions (1 point for each condition): hypertension, obesity, type 2 diabetes, hypercholesterolemia, cancer, and depression).

^6^Self-reported measure of nervousness and/or aggressiveness behavior was reported on a scale from 1 (very low self-perception) to 10 (very high self-perception).

^7^*P*-values for trend were calculated by assigning the median value to each category and treating the resulting variable as continuous.

^8^Recruitment year was referred to the period (years) in which participants were recruited, from the date of the first recruited participant (9/05/2013) to the date of the last recruited participant (10/31/2016).

^9^Total workload of center was measured as the number of participants in the intervention group per center.

^10^Carbohydrate Quality Index was referred to the quality of dietary carbohydrate intake and was constructed upon four carbohydrate quality domains: total dietary fiber intake (g/d), glycemic index, ratio of carbohydrates from whole grains to carbohydrates from total grains (whole grains + refined grains or their products), and ratio of carbohydrates from solid foods to total carbohydrates (solid carbohydrates + liquid carbohydrates). Quartiles of carbohydrate Quality Index (score) were: Q1: <9; Q2: 9-10; Q3: 11-12; Q4: >12.

| **Table s4.** **Analyses only among participants with baseline score <13 points in the active intervention group of the PREDIMED-PLUS trial (n=2,793). Participants with baseline score ≥13 points at baseline were excluded (n=192).** Odds Ratios (OR) and 95% confidence intervals (95% CI) of attaining good adherence^1^ (increasing the score in ≥5 points from baseline) to the MedDiet intervention at 6 and 12 months of follow-up. | | | | | | | |  |
| --- | --- | --- | --- | --- | --- | --- | --- | --- |
|  |  | | **OR (95 % CI) for adherence (increasing ≥5 points)^1^ to the MedDiet intervention (adherent vs. non-adherent) ^2^** | | | | |  |
|  |  | | **6-month follow-up** | |  | **12-month follow-up** | |  |
| Baseline characteristics | **n** | | **Multivariable^3^** | ***p*-value** |  | **Multivariable^3^** | ***p*-value** |  |
| **Socio-demographics** |  | |  |  |  |  |  |  |
| Sex |  | |  |  |  |  |  |  |
| Men | 1458 | | 1.00 (ref) | -- |  | 1.00 (ref) | -- |  |
| Women | 1335 | | 1.23 (0.94-1.60) | 0.127 |  | 1.12 (0.86-1.46) | 0.398 |  |
| Age, years |  | |  |  |  |  |  |  |
| < 65 | 1312 | | 1.00 (ref) | -- |  | 1.00 (ref) | -- |  |
| ≥ 65 | 1481 | | 0.90 (0.72-1.13) | 0.378 |  | 0.82 (0.65-1.03) | 0.085 |  |
| Marital status |  | |  |  |  |  |  |  |
| Married | 2113 | | 1.00 (ref) | -- |  | 1.00 (ref) | -- |  |
| Single | 146 | | **0.57 (0.38-0.86)** | **0.008** |  | 0.81 (0.54-1.21) | 0.296 |  |
| Widowed | 305 | | 0.94 (0.70-1.26) | 0.672 |  | 0.90 (0.66-1.21) | 0.473 |  |
| Others/Missing | 229 | | 0.88 (0.64-1.22) | 0.460 |  | 0.79 (0.57-1.09) | 0.153 |  |
| Attained education level |  | |  |  |  |  |  |  |
| College/university | 583 | | 1.00 (ref) | -- |  | 1.00 (ref) |  |  |
| Secondary | 867 | | 0.97 (0.76-1.24) | 0.805 |  | 0.87 (0.68-1.11) | 0.250 |  |
| Primary or less | 1314 | | 1.07 (0.83-1.37) | 0.612 |  | 0.98 (0.76-1.25) | 0.859 |  |
| Missing | 29 | | 1.71 (0.70-4.20) | 0.239 |  | 0.99 (0.41-2.40) | 0.989 |  |
| Occupation |  | |  |  |  |  |  |  |
| Retired | 1558 | | 1.00 (ref) | -- |  | 1.00 (ref) | -- |  |
| Working | 591 | | 0.91 (0.69-1.19) | 0.484 |  | **0.68 (0.52-0.90)** | **0.007** |  |
| Unemployed or unable to work | 225 | | 0.83 (0.58-1.18) | 0.303 |  | **0.67 (0.47-0.96)** | **0.031** |  |
| Housewife | 402 | | 0.87 (0.65-1.16) | 0.343 |  | 0.90 (0.68-1.20) | 0.480 |  |
| Missing | 17 | | 0.95 (0.31-2.90) | 0.931 |  | 1.10 (0.37-3.34) | 0.860 |  |
| Number of people in household | 2793 | | 0.95 (0.87-1.04) | 0.275 |  | 1.02 (0.94-1.12) | 0.600 |  |
| **Health-related characteristics** |  | |  |  |  |  |  |  |
| ^4^Family history of premature CVD |  | |  |  |  |  |  |  |
| No | 2439 | | 1.00 (ref) | -- |  | 1.00 (ref) | -- |  |
| Yes | 354 | | 1.11 (0.86-1.44) | 0.422 |  | 0.91 (0.70-1.18) | 0.484 |  |
| ^5^Number of chronic conditions |  | |  |  |  |  |  |  |
| ≤ 3 | 2123 | | 1.00 (ref) |  |  | 1.00 (ref) | -- |  |
| > 3 | 670 | | **0.67 (0.54-0.83)** | **< 0.001** |  | **0.75 (0.61-0.94)** | **0.010** |  |
| ^6,7^Self-reported measure of nervousness and/or aggressiveness behavior, score |  | |  |  |  |  |  |  |
| Q1(<4) | 764 | | 1.00 (ref) | -- |  | 1.00 (ref) | -- |  |
| Q2 (4-5) | 1026 | | 0.90 (0.72-1.12) |  |  | 0.97 (0.78-1.20) |  |  |
| Q3 (6) | 328 | | 0.88 (0.65-1.19) |  |  | 1.01 (0.75-1.36) |  |  |
| Q4 (>6) | 675 | | 1.14 (0.90-1.46) | 0.589 |  | 1.31 (1.03-1.68) | 0.084 |  |
| Body weight, (per 5 kg) | 2793 | | 1.02 (0.96-1.09) | 0.489 |  | 1.04 (0.97-1.11) | 0.257 |  |
| Waist circumference (per 5 cm) | 2793 | | 1.03 (0.95-1.11) | 0.525 |  | 0.98 (0.91-1.06) | 0.657 |  |
| SBP (per 5mm Hg) | 2793 | | 1.03 (1.00-1.06) | 0.056 |  | 1.00 (0.97-1.04) | 0.835 |  |
| DBP (per 5mm Hg) | 2793 | | 1.02 (0.96-1.08) | 0.522 |  | 1.02 (0.96-1.07) | 0.525 |  |
| Fasting blood glucose (per 10 mg/dL) | 2793 | | 1.00 (0.97-1.04) | 0.846 |  | 0.99 (0.96-1.03) | 0.650 |  |
| **Study Design Features** |  | |  |  |  |  |  |  |
| ^8^Recruitment year |  | |  |  |  |  |  |  |
| < 1^st^ | 249 | | 1.00 (ref) | -- |  | 1.00 (ref) | -- |  |
| 1^st^-2^nd^ | 713 | | 1.10 (0.78-1.55) | 0.595 |  | **1.62 (1.15-2.28)** | **0.006** |  |
| 2^nd^-3^rd^ | 1413 | | 1.34 (0.96-1.86) | 0.086 |  | **1.73 (1.24-2.40)** | **0.001** |  |
| > 3^rd^ | 418 | | 0.95 (0.65-1.39) | 0.782 |  | 1.33 (0.91-1.95) | 0.142 |  |
| ^9^Total workload of center, participants in intervention group |  | |  |  |  |  |  |  |
| Below median (≤ 120) | 1402 | | 1.00 (ref) | -- |  | 1.00 (ref) | -- |  |
| Above median (>120) | 1391 | | 0.95 (0.78-1.14) | 0.556 |  | 1.10 (0.91-1.33) | 0.311 |  |
| **Lifestyle behavior** |  | |  |  |  |  |  |  |
| Physical activity |  | |  |  |  |  |  |  |
| ^7^METs-min/wk |  | |  |  |  |  |  |  |
| Q1 (<840) | 757 | | 1.00 (ref) | -- |  | 1.00 (ref) | -- |  |
| Q2 (840-1748) | 641 | | **1.31 (1.02-1.69)** |  |  | 1.01 (0.78-1.29) |  |  |
| Q3 (1749-3286) | 697 | | 1.22 (0.94-1.58) |  |  | 0.99 (0.76-1.28) |  |  |
| Q4 (>3286) | 698 | | 0.93 (0.71-1.23) | 0.211 |  | 1.03 (0.78-1.35) | 0.855 |  |
| RAPA test |  | |  |  |  |  |  |  |
| Level 1 (sedentary or under-active) | 535 | | 1.00 (ref) | -- |  | 1.00 (ref) | -- |  |
| Level 2 (under-active regular – light activities) | 1025 | | 0.99 (0.77-1.27) | 0.941 |  | 1.03 (0.81-1.32) | 0.791 |  |
| Level 3 (under-active regular) | 495 | | 1.12 (0.82-1.52) | 0.465 |  | **1.41 (1.04-1.92)** | **0.028** |  |
| Level 4 (active) | 738 | | 1.10 (0.81-1.48) | 0.544 |  | **1.38 (1.02-1.86)** | **0.036** |  |
| ^7^Chair test 30s, repeats |  | |  |  |  |  |  |  |
| Q1 (<12) | 903 | | 1.00 (ref) | -- |  | 1.00 (ref) | -- |  |
| Q2 (12-13) | 615 | | 1.02 (0.80-1.30) |  |  | 1.06 (0.83-1.35) |  |  |
| Q3 (14-16) | 727 | | 1.09 (0.86-1.39) |  |  | 1.16 (0.92-1.48) |  |  |
| Q4 (>16) | 548 | | 0.92 (0.71-1.20) | 0.674 |  | 0.89 (0.69-1.17) | 0.575 |  |
| Smoking status, n(%) |  | |  |  |  |  |  |  |
| Never smokers | 1241 | | 1.00 (ref) | -- |  | 1.00 (ref) | -- |  |
| Current smokers | 387 | | 0.77 (0.58-1.02) | 0.064 |  | **0.71 (0.53-0.94)** | **0.016** |  |
| Former smokers | 1152 | | 0.97 (0.78-1.19) | 0.761 |  | 0.94 (0.76-1.16) | 0.541 |  |
| Missing | 13 | | 0.37 (0.10-1.38) | 0.140 |  | 0.87 (0.25-3.07) | 0.827 |  |
| Alcohol intake other than wine |  | |  |  |  |  |  |  |
| Abstainers | 1403 | | 1.00 (ref) | -- |  | 1.00 (ref) | -- |  |
| ≤ 5 g/d | 738 | | 0.96 (0.78-1.18) | 0.670 |  | 0.94 (0.76-1.15) | 0.529 |  |
| > 5 g/d | 652 | | 0.91 (0.70-1.19) | 0.504 |  | 0.91 (0.70-1.19) | 0.500 |  |
| ^8^Sleeping, hours/d |  | |  |  |  |  |  |  |
| Q1 (<7) | 894 | | 1.00 (ref) | -- |  | 1.00 (ref) | -- |  |
| Q2 (7) | 918 | | 1.03 (0.83-1.28) |  |  | 0.93 (0.75-1.16) |  |  |
| Q3 (8) | 736 | | 0.90 (0.71-1.12) |  |  | 1.05 (0.84-1.32) |  |  |
| Q4 (>8) | 245 | | 1.14 (0.82-1.58) | 0.888 |  | 0.99 (0.71-1.37) | 0.768 |  |
| Self-efficacy for diet modification |  | |  |  |  |  |  |  |
| Little or some | 709 | | 1.00 (ref) | -- |  | 1.00 (ref) | -- |  |
| High | 2084 | | **1.42 (1.16-1.74)** | **< 0.001** |  | **1.61 (1.31-1.96)** | **< 0.001** |  |
| **Total energy and nutrient intake** | |  |  |  |  |  |  | |
| ^7^Total energy intake, kcal/d |  | |  |  |  |  |  |  |
| Q1 (men <2121; women <1881) | 699 | | 1.00 (ref) | -- |  | 1.00 (ref) | -- |  |
| Q2 (men 2121-2482; women: 1881-2215) | 698 | | 0.97 (0.75-1.27) |  |  | 0.96 (0.74-1.25) |  |  |
| Q3 (men 2483-2890; women: 2216-2577) | 699 | | 1.02 (0.77-1.35) |  |  | 1.06 (0.80-1.41) |  |  |
| Q4 (men >2890; women: >2577) | 697 | | 0.88 (0.64-1.21) | 0.442 |  | 0.91 (0.66-1.25) | 0.637 |  |
| Predefined limits of energy intake (Willet), kcal/d |  | |  |  |  |  |  |  |
| Within limits (men 800-4000; women 500-3500) | 2725 | | 1.00 (ref) |  |  | 1.00 (ref) | -- |  |
| Beyond limits (men < 800 or >4000; women <500 or >3500) | 68 | | 0.74 (0.41-1.32) | 0.303 |  | 1.07 (0.59-1.93) | 0.828 |  |
| ^7^Fruit + vegetable consumption, g/d |  | |  |  |  |  |  |  |
| Q1 (men, <469; women <540) | 699 | | 1.00 (ref) | -- |  | 1.00 (ref) | -- |  |
| Q2 (men 469-616; women 540-688) | 698 | | 1.26 (0.97-1.64) |  |  | 1.00 (0.77-1.30) |  |  |
| Q3 (men 617-784; women 689-879) | 699 | | 1.01 (0.76-1.35) |  |  | 0.97 (0.73-1.30) |  |  |
| Q4 (men >784; women >879) | 697 | | 0.89 (0.65-1.22) | 0.204 |  | 0.85 (0.62-1.17) | 0.274 |  |
| ^7^Meat consumption, g/d |  | |  |  |  |  |  |  |
| Q1 (men <116; women <105) | 699 | | 1.00 (ref) | -- |  | 1.00 (ref) | -- |  |
| Q2 (men 116-149; women 105-138) | 698 | | 1.24 (0.97-1.59) |  |  | 1.00 (0.78-1.28) |  |  |
| Q3 (men 150-188; women 139-176) | 700 | | 1.12 (0.87-1.44) |  |  | 1.06 (0.82-1.36) |  |  |
| Q4 (men >188; women >176) | 696 | | 1.05 (0.80-1.37) | 0.974 |  | 0.88 (0.68-1.16) | 0.411 |  |
| ^7^Baseline Dietary fat intake, % E, mean (SD) |  | |  |  |  |  |  |  |
| Q1 (<35) | 699 | | 1.00 (ref) | -- |  | 1.00 (ref) | -- |  |
| Q2 (35-38) | 698 | | 1.20 (0.94-1.54) |  |  | 1.06 (0.83-1.36) |  |  |
| Q3 (39-43) | 698 | | 1.07 (0.83-1.37) |  |  | 0.93 (0.72-1.19) |  |  |
| Q4 (>43) | 698 | | 1.25 (0.96-1.63) | 0.174 |  | 1.09 (0.84-1.42) | 0.716 |  |
| ^7^Fiber intake, g/d |  | |  |  |  |  |  |  |
| Q1 (men <20; women < 21) | 699 | | 1.00 (ref) | -- |  | 1.00 (ref) | -- |  |
| Q2 (men 20-24; women 21-25) | 698 | | 1.07 (0.81-1.43) |  |  | **1.34 (1.01-1.79)** |  |  |
| Q3 (men 25-30; women 26-32) | 699 | | **1.46 (1.03-2.06)** |  |  | **1.46 (1.03-2.07)** |  |  |
| Q4 (men >30 women >32) | 697 | | **1.78 (1.15-2.76)** | **0.007** |  | **1.63 (1.05-2.53)** | 0.053 |  |
| ^7,10^Carbohydrate Quality Index |  | |  |  |  |  |  |  |
| Q1 (low) | 922 | | 1.00 (ref) | -- |  | 1.00 (ref) | -- |  |
| Q2 | 775 | | 0.88 (0.69-1.11) |  |  | 0.88 (0.69-1.12) |  |  |
| Q3 | 606 | | 0.81 (0.61-1.07) |  |  | 0.86 (0.64-1.14) |  |  |
| Q4 (high) | 490 | | 0.82 (0.58-1.15) | 0.181 |  | 0.85 (0.60-1.20) | 0.319 |  |

Abbreviations: CVD, cardiovascular disease; DBP, diastolic blood pressure; E, energy; MedDiet, Mediterranean diet; MET, metabolic equivalent; Q, quartile; RAPA, rapid assessment of physical activity; SBP, systolic blood pressure.

Data available in the intervention group of the PREDIMED-PLUS trial (n = 2793); for marital status (n = 10 missing); for attained education level (n = 29 missing); for occupation (n =17 missing); for number of people in household (n = 5 missing); for self-reported measure of nervousness and/or aggressiveness behavior (n = 26 missing); for SBP (n = 21 missing); for DBP (n = 21 missing); for fasting blood glucose (n = 39 missing); for RAPA test (n = 1 missing); for smoking status (n = 13 missing); for sleeping hours (n = 36 missing).

^1^Adherence to Mediterranean diet was evaluated using a 17-point scale of adherence to an energy-reduced MedDiet questionnaire (1 point for each item). Participants with an increase of ≥5 points from baseline to follow-up were classified in the “*adherent group*”. Participants with ≥13 points at baseline were exclude from the analysis.

^2^ORs <1 was referred as poorer adherence and ORs >1 was referred as better adherence.

^3^Multivariable model implied multivariable-adjusted logistic regression, adjusted for all characteristics displayed in Table 2 with the addition of the 17-item energy-reduced MedDiet score

^4^Family history of premature CVD was defined as any immediate family member deceased younger than 55 years for men and 65 years for women.

^5^Number of chronic conditions was calculated by summing the following chronic conditions (1 point for each condition): hypertension, obesity, type 2 diabetes, hypercholesterolemia, cancer, and depression).

^6^Self-reported measure of nervousness and/or aggressiveness behavior was reported on a scale from 1 (very low self-perception) to 10 (very high self-perception).

^7^P-values for trend were calculated by assigning the median value to each category and treating the resulting variable as continuous.

^8^Recruitment year was referred to the period (years) in which participants were recruited, from the date of the first recruited participant (9/05/2013) to the date of the last recruited participant (10/31/2016).

^9^Total workload of center was measured as the number of participants in the intervention group per center.

^10^Carbohydrate Quality Index was referred to the quality of dietary carbohydrate intake and was constructed upon four carbohydrate quality domains: total dietary fiber intake (g/d), glycemic index, ratio of carbohydrates from whole grains to carbohydrates from total grains (whole grains + refined grains or their products), and ratio of carbohydrates from solid foods to total carbohydrates (solid carbohydrates + liquid carbohydrates). Quartiles of carbohydrate Quality Index (score) were: Q1: <9; Q2: 9-10; Q3: 11-12; Q4: >12.

| **Table s5.** **Analyses using an alternate definition of good adherence^1^: score >12 points to the MedDiet intervention.** Odds Ratios (OR) and 95% confidence intervals (95% CI) of attaining good adherence^1^ (score >12 points) to the MedDiet intervention at 6 and 12 months of follow-up in the active intervention group of the PREDIMED-PLUS trial^1^ (n=2,985) | | | | | | |
| --- | --- | --- | --- | --- | --- | --- |
|  |  | **OR (95 % CI) for adherence (score >12 points)^1^ to the MedDiet intervention (adherent vs. non-adherent) ^2^** | | | | |
|  |  | **6-month follow-up** | |  | **12-month follow-up** | |
| Baseline characteristics | **n** | **Multivariable^3^** | ***p*-value** |  | **Multivariable^3^** | ***p*-value** |
| **Socio-demographics** |  |  |  |  |  |  |
| Sex |  |  |  |  |  |  |
| Men | 1540 | 1.00 (ref) | -- |  | 1.00 (ref) | -- |
| Women | 1445 | 1.29 (1.02-1.63) | 0.037 |  | 1.03 (0.81-1.30) | 0.830 |
| Age, years |  |  |  |  |  |  |
| < 65 | 1404 | 1.00 (ref) | -- |  | 1.00 (ref) | -- |
| ≥ 65 | 1581 | 0.87 (0.71-1.06) | 0.173 |  | 0.89 (0.72-1.09) | 0.255 |
| Marital status |  |  |  |  |  |  |
| Married | 2253 | 1.00 (ref) | -- |  | 1.00 (ref) | -- |
| Single | 160 | 0.71 (0.50-1.01) | 0.057 |  | 1.10 (0.77-1.57) | 0.619 |
| Widowed | 325 | 0.91 (0.69-1.19) | 0.490 |  | 1.11 (0.85-1.46) | 0.453 |
| Others/Missing | 247 | 0.82 (0.61-1.10) | 0.176 |  | 0.76 (0.57-1.02) | 0.069 |
| Attained education level |  |  |  |  |  |  |
| College/university | 642 | 1.00 (ref) | -- |  | 1.00 (ref) |  |
| Secondary | 913 | 0.94 (0.76-1.18) | 0.616 |  | 0.92 (0.74-1.15) | 0.449 |
| Primary or less | 1401 | 0.87 (0.70-1.09) | 0.233 |  | 0.97 (0.78-1.21) | 0.793 |
| Missing | 29 | 1.35 (0.57-3.23) | 0.496 |  | 1.49 (0.62-3.61) | 0.375 |
| Occupation |  |  |  |  |  |  |
| Retired | 1665 | 1.00 (ref) | -- |  | 1.00 (ref) | -- |
| Working | 628 | **0.71 (0.56-0.91)** | **0.006** |  | **0.75 (0.59-0.96)** | **0.024** |
| Unemployed or unable to work | 237 | 0.84 (0.61-1.16) | 0.288 |  | 0.76 (0.55-1.05) | 0.098 |
| Housewife | 435 | 0.88 (0.68-1.15) | 0.353 |  | 0.99 (0.77-1.29) | 0.958 |
| Missing | 20 | 1.20 (0.45-3.16) | 0.714 |  | 1.78 (0.61-5.19) | 0.292 |
| Number of people in household | 2985 | 0.93 (0.86-1.01) | 0.078 |  | 1.02 (0.94-1.11) | 0.641 |
| **Health-related characteristics** |  |  |  |  |  |  |
| ^4^Family history of premature CVD |  |  |  |  |  |  |
| No | 2606 | 1.00 (ref) | -- |  | 1.00 (ref) | -- |
| Yes | 379 | 1.11 (0.88-1.40) | 0.375 |  | 0.88 (0.70-1.11) | 0.297 |
| ^5^Number of chronic conditions |  |  |  |  |  |  |
| ≤ 3 | 2267 | 1.00 (ref) | -- |  | 1.00 (ref) | -- |
| > 3 | 718 | **0.69 (0.57-0.84)** | **< 0.001** |  | 0.83 (0.68-1.01) | 0.060 |
| ^6,7^Self-reported measure of nervousness and/or aggressiveness behavior, score |  |  |  |  |  |  |
| Q1(<4) | 816 | 1.00 (ref) | -- |  | 1.00 (ref) | -- |
| Q2 (4-5) | 1096 | 0.95 (0.78-1.16) |  |  | 0.93 (0.76-1.13) |  |
| Q3 (6) | 347 | 1.00 (0.76-1.31) |  |  | 0.99 (0.75-1.30) |  |
| Q4 (>6) | 726 | 1.14 (0.92-1.42) | 0.380 |  | 1.18 (0.95-1.47) | 0.275 |
| Body weight, (per 5 kg) | 2985 | 1.05 (0.99-1.11) | 0.127 |  | 1.05 (0.99-1.11) | 0.085 |
| Waist circumference (per 5 cm) | 2985 | 1.00 (0.93-1.08) | 0.976 |  | 0.94 (0.88-1.01) | 0.103 |
| SBP (per 5mm Hg) | 2985 | 1.01 (0.99-1.04) | 0.318 |  | 1.00 (0.97-1.03) | 0.917 |
| DBP (per 5mm Hg) | 2985 | 1.03 (0.98-1.08) | 0.216 |  | 1.03 (0.98-1.08) | 0.215 |
| Fasting blood glucose (per 10 mg/dL) | 2985 | 1.01 (0.98-1.04) | 0.450 |  | 1.01 (0.98-1.04) | 0.670 |
| **Study Design Features** |  |  |  |  |  |  |
| ^8^Recruitment year |  |  |  |  |  |  |
| < 1^st^ | 268 | 1.00 (ref) | -- |  | 1.00 (ref) | -- |
| 1^st^-2^nd^ | 761 | 1.16 (0.85-1.57) | 0.351 |  | **1.48 (1.09-2.00)** | **0.012** |
| 2^nd^-3^rd^ | 1506 | **1.34 (1.00-1.80)** | **0.048** |  | **1.48 (1.10-1.98)** | **0.009** |
| > 3^rd^ | 450 | 1.03 (0.73-1.44) | 0.870 |  | 1.31 (0.93-1.83) | 0.121 |
| ^9^Total workload of center, participants in intervention group |  |  |  |  |  |  |
| Below median (≤ 128) | 1498 | 1.00 (ref) | -- |  | 1.00 (ref) | -- |
| Above median (>128) | 1487 | **0.71 (0.60-0.83)** | **< 0.001** |  | **0.80 (0.68-0.95)** | **0.008** |
| **Lifestyle behavior** |  |  |  |  |  |  |
| Physical activity |  |  |  |  |  |  |
| ^7^METs-min/wk |  |  |  |  |  |  |
| Q1 (<840) | 778 | 1.00 (ref) | -- |  | 1.00 (ref) | -- |
| Q2 (840-1818) | 720 | **1.26 (1.01-1.58)** |  |  | 1.01 (0.80-1.26) |  |
| Q3 (1819-3356) | 762 | 0.96 (0.76-1.21) |  |  | 1.02 (0.81-1.28) |  |
| Q4 (>3356) | 725 | 0.96 (0.75-1.24) | 0.291 |  | 0.98 (0.76-1.26) | 0.849 |
| RAPA test |  |  |  |  |  |  |
| Level 1 (sedentary or under-active) | 556 | 1.00 (ref) | -- |  | 1.00 (ref) | -- |
| Level 2 (under-active regular – light activities) | 1079 | 1.06 (0.85-1.33) | 0.596 |  | 1.19 (0.96-1.49) | 0.119 |
| Level 3 (under-active regular) | 531 | 1.26 (0.96-1.65) | 0.097 |  | **1.52 (1.16-2.01)** | 0.003 |
| Level 4 (active) | 819 | **1.47 (1.13-1.93)** | **0.005** |  | **1.52 (1.16-1.99)** | **0.002** |
| ^7^Chair test 30s, repeats |  |  |  |  |  |  |
| Q1 (<12) | 962 | 1.00 (ref) | -- |  | 1.00 (ref) | -- |
| Q2 (12-13) | 665 | 1.09 (0.88-1.35) |  |  | 1.14 (0.92-1.42) |  |
| Q3 (14-16) | 762 | 1.14 (0.92-1.41) |  |  | 1.17 (0.94-1.44) |  |
| Q4 (>16) | 596 | 0.96 (0.76-1.22) | 0.815 |  | 1.07 (0.84-1.36) | 0.561 |
| Smoking status, n(%) |  |  |  |  |  |  |
| Never smokers | 1337 | 1.00 (ref) | -- |  | 1.00 (ref) | -- |
| Current smokers | 397 | **0.74 (0.58-0.96)** | **0.024** |  | **0.73 (0.57-0.95)** | **0.017** |
| Former smokers | 1237 | 0.98 (0.81-1.18) | 0.801 |  | 1.04 (0.86-1.25) | 0.721 |
| Missing | 14 | 0.54 (0.17-1.70) | 0.293 |  | 0.53 (0.17-1.66) | 0.278 |
| Alcohol intake other than wine |  |  |  |  |  |  |
| Abstainers | 1494 | 1.00 (ref) | -- |  | 1.00 (ref) | -- |
| ≤ 5 g/d | 789 | 0.89 (0.74-1.07) | 0.212 |  | 0.96 (0.80-1.16) | 0.678 |
| > 5 g/d | 702 | 0.96 (0.76-1.21) | 0.716 |  | 0.95 (0.75-1.21) | 0.685 |
| ^7^Sleeping, hours/d |  |  |  |  |  |  |
| Q1 (<7) | 964 | 1.00 (ref) | -- |  | 1.00 (ref) | -- |
| Q2 (7) | 984 | 0.92 (0.76-1.11) |  |  | 0.97 (0.80-1.17) |  |
| Q3 (8) | 777 | 0.98 (0.80-1.20) |  |  | 0.92 (0.75-1.13) |  |
| Q4 (>8) | 260 | 1.06 (0.79-1.43) | 0.859 |  | 1.02 (0.76-1.37) | 0.677 |
| Self-efficacy for diet modification |  |  |  |  |  |  |
| Little or some | 738 | 1.00 (ref) | -- |  | 1.00 (ref) | -- |
| High | 2247 | **1.30 (1.08-1.56)** | **0.005** |  | **1.30 (1.09-1.56)** | **0.004** |
| **Total energy and nutrient intake** |  |  |  |  |  |  |
| ^7^Total energy intake, kcal/d |  |  |  |  |  |  |
| Q1 (men <2121; women <1889) | 747 | 1.00 (ref) | -- |  | 1.00 (ref) | -- |
| Q2 (men 2121-2477; women: 1889-2214) | 746 | 0.98 (0.78-1.24) |  |  | 1.04 (0.82-1.31) |  |
| Q3 (men 2478-2885; women: 2215-2564) | 746 | 0.99 (0.77-1.27) |  |  | 1.08 (0.84-1.39) |  |
| Q4 (men >2885; women: >2564) | 746 | 0.83 (0.62-1.10) | 0.187 |  | 0.94 (0.70-1.25) | 0.640 |
| Predefined limits of energy intake (Willet), kcal/d |  |  |  |  |  |  |
| Within limits (men 800-4000; women 500-3500) | 2913 | 1.00 (ref) |  |  | 1.00 (ref) | -- |
| Beyond limits (men < 800 or >4000; women <500 or >3500) | 72 | 0.69 (0.41-1.16) | 0.159 |  | 0.83 (0.49-1.39) | 0.473 |
| ^7^Fruit + vegetable consumption, g/d |  |  |  |  |  |  |
| Q1 (men, <473; women <544) | 747 | 1.00 (ref) | -- |  | 1.00 (ref) | -- |
| Q2 (men 473-624; women 544-69) | 746 | **1.27 (1.01-1.60)** |  |  | 1.09 (0.86-1.38) |  |
| Q3 (men 625-795; women 699-886) | 746 | 1.00 (0.78-1.30) |  |  | 0.87 (0.67-1.13) |  |
| Q4 (men >795; women >886) | 746 | 1.07 (0.81-1.42) | 0.987 |  | 0.79 (0.59-1.05) | 0.045 |
| ^7^Meat consumption, g/d |  |  |  |  |  |  |
| Q1 (men <114; women <105) | 747 | 1.00 (ref) | -- |  | 1.00 (ref) | -- |
| Q2 (men 114-147; women 105-137) | 746 | **1.29 (1.03-1.62)** |  |  | 1.12 (0.90-1.41) |  |
| Q3 (men 148-188; women 138-174) | 747 | 0.99 (0.79-1.24) |  |  | 0.97 (0.77-1.21) |  |
| Q4 (men >188; women >174) | 745 | 0.91 (0.72-1.16) | 0.160 |  | 0.95 (0.75-1.20) | 0.426 |
| ^7^Baseline Dietary fat intake, % E, mean (SD) |  |  |  |  |  |  |
| Q1 (<35) | 747 | 1.00 (ref) | -- |  | 1.00 (ref) | -- |
| Q2 (35-39) | 746 | 1.20 (0.96-1.50) |  |  | 0.93 (0.75-1.17) |  |
| Q3 (40-43) | 746 | 1.16 (0.93-1.46) |  |  | 1.05 (0.84-1.32) |  |
| Q4 (>43) | 746 | 1.22 (0.96-1.55) | 0.129 |  | 1.07 (0.84-1.36) | 0.422 |
| ^7^Fiber intake, g/d |  |  |  |  |  |  |
| Q1 (men <20; women < 21) | 747 | 1.00 (ref) | -- |  | 1.00 (ref) | -- |
| Q2 (men 20-24; women 21-25) | 746 | 1.09 (0.84-1.41) |  |  | 1.29 (1.00-1.67) |  |
| Q3 (men 25-30; women 26-32) | 746 | 1.28 (0.94-1.75) |  |  | **1.53 (1.12-2.10)** |  |
| Q4 (men >30 women >32) | 746 | **1.66 (1.12-2.47)** | **0.007** |  | **1.79 (1.20-2.67)** | **0.007** |
| ^7,10^Carbohydrate Quality Index |  |  |  |  |  |  |
| Q1 (low) | 981 | 1.00 (ref) | -- |  | 1.00 (ref) | -- |
| Q2 | 803 | 1.04 (0.84-1.30) |  |  | 0.93 (0.75-1.15) |  |
| Q3 | 682 | 1.05 (0.81-1.35) |  |  | 0.90 (0.70-1.17) |  |
| Q4 (high) | 519 | 1.00 (0.73-1.38) | 0.880 |  | 0.94 (0.68-1.30) | 0.565 |

Abbreviations: CVD, cardiovascular disease; DBP, diastolic blood pressure; E, energy; M, months; MedDiet, Mediterranean diet; MET, metabolic equivalent; Q, quartile; RAPA, rapid assessment of physical activity; SBP, systolic blood pressure.

Data available in the intervention group of the PREDIMED-PLUS trial (n = 2985); for marital status (n = 10 missing); for attained education level (n = 29 missing); for occupation (n = 20 missing); for number of people in household (n = 5 missing); for self-reported measure of nervousness and/or aggressiveness behavior (n = 26 missing); for SBP (n = 23 missing); for DBP (n = 23 missing); for fasting blood glucose (n = 42 missing); for RAPA test (n = 1 missing); for smoking status (n = 14 missing); for sleeping hours (n = 38 missing).

^1^Adherence to Mediterranean diet was evaluated using a 17-point scale of adherence to an energy-reduced MedDiet questionnaire (1 point for each item). Participants with >12 points were classified in the “*adherent group*”.

^2^ORs <1 was referred as poorer adherence and ORs >1 was referred as better adherence.

^3^Multivariable model implied multivariable-adjusted logistic regression, adjusted for all characteristics displayed in Table 2 with the addition of the 17-item energy-reduced MedDiet score.

^4^Family history of premature CVD was defined as any immediate family member deceased younger than 55 years for men and 65 years for women.

^5^Number of chronic conditions was calculated by summing the following chronic conditions (1 point for each condition): hypertension, obesity, type 2 diabetes, hypercholesterolemia, cancer, and depression).

^6^Self-reported measure of nervousness and/or aggressiveness behavior was reported on a scale from 1 (very low self-perception) to 10 (very high self-perception).

^7^P-values for trend were calculated by assigning the median value to each category and treating the resulting variable as continuous.

^8^Recruitment year was referred to the period (years) in which participants were recruited, from the date of the first recruited participant (9/05/2013) to the date of the last recruited participant (10/31/2016).

^9^Total workload of center was measured as the number of participants in the intervention group per center.

^10^Carbohydrate Quality Index was referred to the quality of dietary carbohydrate intake and was constructed upon four carbohydrate quality domains: total dietary fiber intake (g/d), glycemic index, ratio of carbohydrates from whole grains to carbohydrates from total grains (whole grains + refined grains or their products), and ratio of carbohydrates from solid foods to total carbohydrates (solid carbohydrates + liquid carbohydrates). Quartiles of carbohydrate Quality Index (score) were: Q1: <9; Q2: 9-10; Q3: 11-12; Q4: >12.

**FIGURES**

**Figure s1.** Outcome definition of adherent and non-adherent participants on the active group of the PREDIMED-PLUS trial.


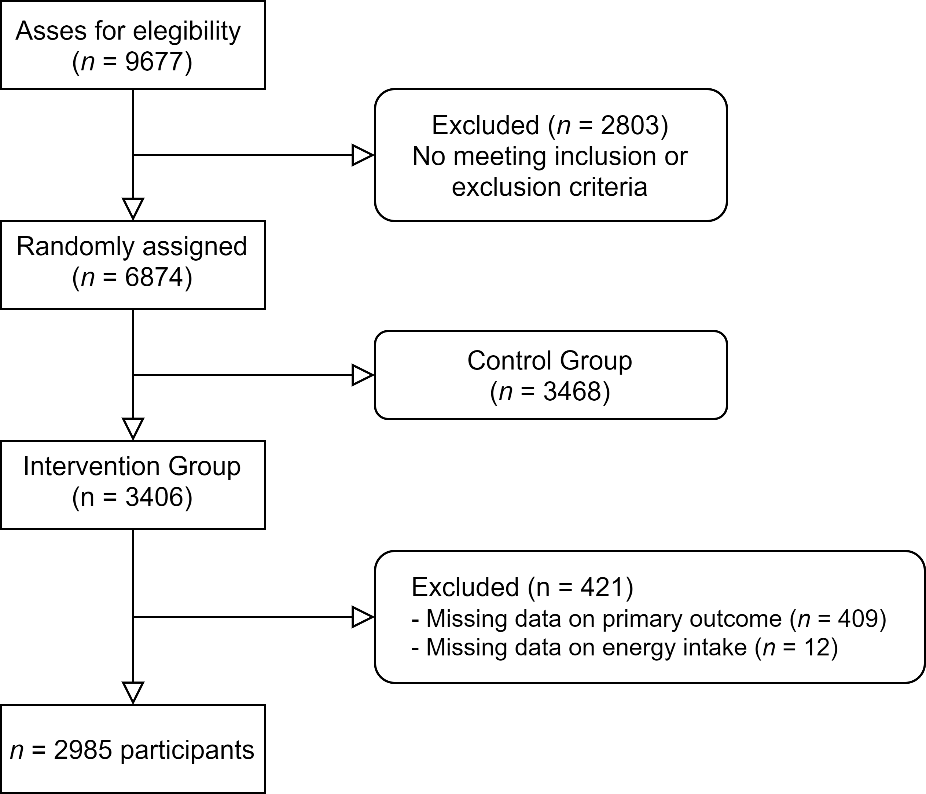


**Figure s2.** Flow chart of the participants of the study. The PREDIMED-Plus trial.

**Sources of Support (Grant Numbers)**

Coordinated by J.S.-S. and J.V., including the following projects: PI13/00673, PI13/00492, PI13/00272, PI13/01123, PI13/00462, PI13/00233, PI13/02184, PI13/00728, PI13/01090, PI13/01056, PI14/01722, PI14/00636, PI14/00618, PI14/00696, PI14/01206, PI14/01919, PI14/00853, PI14/01374, PI14/00972, PI14/00728, PI14/01471, PI16/00473, PI16/00662, PI16/01873, PI16/01094, PI16/00501, PI16/00533, PI16/00381, PI16/00366, PI16/01522, PI16/01120, PI17/00764, PI17/01183, PI17/00855, PI17/01347, PI17/00525, PI17/01827, PI17/00532, PI17/00215, PI17/01441, PI17/00508, PI17/01732, PI17/00926, PI19/00957, PI19/00386, PI19/00309, PI19/01032, PI19/00576, PI19/00017, PI19/01226, PI19/00781, PI19/01560, PI19/01332, PI20/01802, PI20/00138, PI20/01532, PI20/00456, PI20/00339, PI20/00557, PI20/00886, and PI20/01158; the Recercaixa grant (2013ACUP00194); grants from the Consejería de Salud de la Junta de Andalucía (PI0458/2013, PS0358/2016, and PI0137/2018), grants from the Generalitat Valenciana (Consellería de Innovación, Ciencia y Sociedad Digital; PROMETEO-17/2017), The International Nut & Dried Fruit Council – FESNAD (Long-term effects of an energy-restricted Mediterranean diet on mortality and cardiovascular disease 2014 –2015, No. 201302) [M.A.M.-G.]; J.K. was supported through "FOLIUM" program within the FUTURMed project. Talent for the medicine within the future from the Fundación Instituto de Investigación Sanitaria Illes Balears (financed by 2017 annual plan of the sustainable tourism tax and at 50% with charge to the ESF Operational Program 2014-2020 of the Balearic Islands). J.S.-S. is partially supported by ICREA under the ICREA Academia programme. Food companies Hojiblanca (Lucena, Spain) and Patrimonio Comunal Olivarero (Madrid, Spain) donated extra virgin olive oil, and the Almond Board of California (Modesto, CA), American Pistachio Growers (Fresno, CA), and Paramount Farms (Wonderful Company, LLC, Los Angeles, CA) donated nuts. JFG-G received Contratos Predoctorales de Formación en Investigación en Salud (PFIS FI17/00255) from Acción Estratégica en Salud program (AES) from ISCIII.

**List of PREDIMED-Plus study investigators**

**Rovira i Virgili University, Department of Biochemistry and Biotechnology, Human Nutrition Unit, University Hospital of Sant Joan de Reus, Pere Virgili Institute for Health Research, Reus, Spain:** R. Pedret Llaberia, R. Gonzalez, R. Sagarra Álamo, F. París Palleja, J. Balsells, J.M. Roca, T. Basora Gallisa, J. Vizcaino, P. Llobet Alpizarte, C. Anguera Perpiñá, M. Llauradó Vernet, C. Caballero, M. Garcia Barco, M.D. Morán Martínez, J. García Rosselló, A. Del Pozo, C. Poblet Calaf, P. Arcelin Zabal, X. Floresví, M. Ciutat Benet, A. Palau Galindo, J.J. Cabré Vila, F. Dolz Andrés, M. Soler, M. Gracia Vidal, J. Vilalta J. Boj Casajuana, M. Ricard, F. Saiz, A. Isach, M. Sanchez Marin Martinez, E. Granado Font, C, Lucena Luque, C. Mestres Sola, N. Babio, N. Becerra-Tomás, J. Basora, G. Mena-Sánchez, L. Barrubés Piñol, N. Rosique-Esteban, S. Chig, I. Abellán Cano, V. Ruiz García, C. Gomez-Martinez, L. Lopez-Gonzalez, A. Salas-Huetos, I. Paz-Graniel, J. Roig Vallverdú, C. Miñana Garcia, L. Sánchez Niembro, P. Hernandez-Alonso, S. Canudas, A. Díaz-López, M. Mendoza Herrera, S. Manzanedo, J. Muralidharan, A. Atzeni, C. Valle. M, Fernández de la Puente, T. Garcidueñas-Fimbres.

**Department of Preventive Medicine and Public Health, University of Navarra-Navarra Institute for Health Research (IdiSNA), Pamplona, Spain:** M. Ruiz-Canela, E. Toledo, P. Buil-Cosiales, Z. Vázquez, C. Razquin, M. Bes-Rastrollo, A. Gea, A. Sanchez Tainta, B. SanJulian Aranguren, E. Goñi, L. Goñi, M.J. Cobo, A. Rico-Campa, F.J. Basterra Gortari, A. Garcia Arellano, J. Diez-Espino, O. Lecea-Juarez, J. Carlos Cenoz-Osinaga, I. Alvarez-Alvarez, M.C. Sayon-Orea, C.I. Fernandez-Lázaro, L. Ruiz-Estigarribia, J. Bartolome-Resano, A. Sola-Larraza (†), E. Lozano-Oloriz, B. Cano-Valles, S. Eguaras, E. Pascual Roquet-Jalmar, I. Galilea-Zabalza, H. Lancova, R. Ramallal, M.L. Garcia-Perez, V. Estremera-Urabayen, M.J. Ariz-Arnedo, C. Hijos-Larraz, C. Fernandez-Alfaro, B. Iñigo-Martinez, R. Villanueva Moreno, S. Martin-Almendros, L. Barandiaran-Bengoetxea, C. Fuertes-Goñi, A. Lezaun-Indurain, M.J. Guruchaga-Arcelus, O. Olmedo-Cruz, L. Escriche-Erviti, R. Ansorena-Ros, R. Sanmatin-Zabaleta, J. Apalategi-Lasa, J. Villanueva-Telleria, M.M. Hernández-Espinosa, L. Herrera-Valdez, L. Dorronsoro-Dorronsoro, Lourdes Echeverria-Lizarraga (†), J.A. Cabeza-Beunza, P. Fernández-Urretavizcaya, P. Gascó-García, C. Royo-Jimenez, J. Moran-Pí, F. Salazar-Fernández, F.J. Chasco-Ros, F. Cortés-Ugalde, J.J. Jurio-Burgui, P. Pascual-Pascual, A.I. Rodríguez-Ezpeleta, M. Esparza-Cáceres, C. Arroyo-Azpa, M. Rodríguez-Sanz de Galdeano, T. Forcen-Alonso, M. Armendariz-Marcotegui, A. Brugos-Larumbe, A. Arillo, B. López-Aisa.

**Department of Preventive Medicine, University of Valencia, University Jaume I, Conselleria de Sanitat de la Generalitat Valenciana, Valencia, Spain:** J.I. González, J.V. Sorlí, O. Portolés, R. Fernández-Carrión, C. Ortega-Azorín, R. Barragán, E.M. Asensio, O. Coltell, R. Martínez-Lacruz, I. Giménez‐Alba, C. Sáiz, R. Osma, E. Férriz, I. González-Monje, P. Guillém-Sáiz, F. Giménez-Fernández, L. Quiles, P. Carrasco, A. Carratalá-Calvo, C. Valero-Barceló, C. Mir, S. Sánchez-Navarro, J. Navas, I. González-Gallego, L. Bort-Llorca, L. Pérez-Ollero, M. Giner-Valero, R. Monfort-Sáez, J. Nadal-Sayol, V. Pascual-Fuster, M. Martínez-Pérez, C. Riera, M.V. Belda, A. Medina, E. Miralles, M.J. Ramírez-Esplugues, M. Rojo-Furió, G. Mattingley, M.A. Delgado, M.A. Pages, Y Riofrío, L. Abuomar, N. Blasco-Lafarga, R. Tosca, L. Lizán, A.M Valcarce, M.D. Medina, S. de Valcárcel, N. Tormo, O. Felipe-Román, S. Lafuente, E.I. Navío, G. Aldana, J.V. Crespo, J.L. Llosa, L. González-García, R. Raga-Marí.

**Cardiovascular Risk and Nutrition Research Group, Endocrinology Service, Neurosciences Programme, Clinical Research Unit at the Hospital del Mar Medical Research Institute (IMIM), Barcelona. Medicine Departament, Universitat Autònoma de Barcelona, Barcelona, Spain**: M. Fitó, O. Castañer, M.A. Muñoz, M.D. Zomeño, A. Hernaéz, L. Torres, M. Quifer, R. Llimona, G Freixer, KA. Pérez-Vega, M. Farràs, R. Elosua, J. Vila, I. Subirana, S. Pérez, A. Goday, J.J. Chillaron Jordan, J.A. Flores Lerroux, D. Benaiges Boix, G. Llauradó, M. Farré, E. Menoyo, A. Aldea-Perona, M. Pérez-Otero, D. Muñoz-Aguayo, S. Gaixas, G. Blanchart, A. Sanllorente, M. Soria, J. Valussi, A. Cuenca, L. Forcano, A. Pastor, A. Boronat, S. Tello, M. Cabañero, L. Franco, H. Schröder, R. De la Torre, C. Medrano, J. Bayó, M.T. García, V. Robledo, P. Babi, E. Canals, N. Soldevila, L. Carrés, C. Roca, M.S. Comas, G. Gasulla, X. Herraiz, A. Martínez, E. Vinyoles, J.M. Verdú, M. Masague Aguade, E. Baltasar Massip, M. López Grau, M. Mengual, V. Moldon, M. Vila Vergaz, R. Cabanes Gómez, Ciurana, M. Gili Riu, A. Palomeras Vidal, F Peñas F, A Raya, M.A. Sebastian, M. Valls, J. Guerrero, M. Marne, E. Minguella, M. Montenegro, A. Sala, M.R. Senan, N. Talens, N. Vera.

**Nutritional Epidemiology Unit, Miguel Hernandez University, ISABIAL-FISABIO, Alicante, Spain:** J. Vioque, M. García-de-la-Hera, S. Gonzalez-Palacios, L. Torres-Collado, L. Compañ-Gabucio, A. Oncina-Canovas, L. Notario-Barandiaran, D. Orozco-Beltran, S. Pertusa Martínez, A. Asencio, I. Candela-García, J.M. Zazo, D. Vivancos Aparicio, N. Fernández-Brufal, J. Román Maciá, F. Ortiz Díaz, M. García Muñoz, C. Barceló, E. Martínez-García, M Damaj-Hamieh, M.C. Martínez Vergara, M.A. Sempere Pascual, S.J. Miralles Gisbert, A. González Botella, C.M. López García, R. Valls Enguix, N. Gómez Bellvert, V. Martínez Avilés, R. Lloret Macián, A. Pastor Morel, M. Mayor-Llorca, J.J. Ballester Baixauli, G. Notario García, M.A. Belmar-Bueno, E.P. Cases Pérez, C. Tercero Maciá, L.A. Mira Castejón, J. Torregrosa García, C. Pastor Polo, E. Puig Agulló, M.V. Hernándis Marsán, M.J. González Fajardo, I. Hervella Durantez, M.C. Latorre Use, A. Bernabé Casanova, F. Medina Ruzafa, E. Robledano, I. Vilanova Martínez, A. Molina Santiago.

**Hospital Son Espases (HUSE) and Institute for Health Research Illes Balears (IdISBa), Palma de Mallorca, Spain:** M. Fiol, M. Moñino, A. Colom, J. Konieczna, M. Morey, A.M. Galmés-Panadés, M.A. Martín, E. Rayó, J. Llobera, J. Fernández-Palomeque, E. Fortuny, M. Noris, L. López, X. Rosselló, S. Munuera, F. Tomás, F. Fiol, A. Jover, J.M. Janer, C. Vallespir, I. Mattei, N. Feuerbach, M. del Mar Sureda, S. Vega, L. Quintana, A. Fiol, M. Amador, S. González, J. Coll, A. Moyá, T. Piqué Sistac, M.D. Sanmartín Fernández, M.C. Piña Valls, M.A. Llorente San Martín, J. Pou Bordoy.

**Department of Nutrition, Food Sciences, and Physiology, Center for Nutrition Research, University of Navarra, Pamplona, Spain:** I. Abete, I. Cantero, C. Cristobo, I. Ibero-Baraibar, M. Zulet, J. Ágreda-Peiró, M.D. Lezáun-Burgui, N. Goñi-Ruiz, R. Bartolomé-Resano, E. Cano-Cáceres, T. Elcarte-López, E. Echarte-Osacain, B. Pérez-Sanz, I. Blanco-Platero, A. Andueza- Azcárate, A. Gimeno-Aznar, E. Ursúa-Sesma, B. Ojeda-Bilbao, J. Martinez-Jarauta, L. Ugalde-Sarasa, B. Rípodas-Echarte, M.V. Güeto-Rubio, C. Napal-Lecumberri, MD Martínez-Mazo, E Arina-Vergara, A. Parra-Osés, F. Artal-Moneva, F. Bárcena-Amigo, F. Calle-Irastoza, J. Abad-Vicente, J.I. Armendáriz-Artola, P. Iñigo-Cibrian, J. Escribano-Jarauta, J. Ulibarri-delportillo, B. Churio-Beraza, Y. Monzón-Martínez, E. Madoz-Zubillaga, C. Arroniz.

**University of Málaga and Institute of Biomedical Research in Malaga (IBIMA), Málaga, Spain:** F.J. Barón-López, J.C. Fernández García, N. Pérez-Farinós, N. Moreno-Morales, M. del C. Rodríguez-Martínez, J. Pérez-López, J.C. Benavente-Marín, E. Crespo Oliva, E. Contreras Fernández, F.J. Carmona González, R. Carabaño Moral, S. Torres Moreno, M.V. Martín Ruíz, M. Alcalá Cornide, V. Fuentes Gómez.

**Lipids and Atherosclerosis Unit, Department of Internal Medicine, Maimonides Biomedical Research Institute of Cordoba (IMIBIC), Reina Sofia University Hospital, University of Cordoba, Cordoba, Spain:** J. López-Miranda, A. Garcia-Rios, J. Criado García, A.I. Jiménez Morales, A. Ortiz Morales, J.D. Torres Peña, F.J. Gómez Delgado, J.F. Alcalá, A. León Acuña, A.P. Arenas Larriva, F. Rodríguez Cantalejo, J. Caballero Villaraso, I. Nieto Eugenio, P. Coronado Carvajal, M.C del Campo Molina, P.J. Peña Orihuela, I. Perez Corral, G. Quintana Navarro.

**Department of Internal Medicine, Institut d’Investigacions Biomèdiques August Pi i Sunyer (IDIBAPS), Hospital Clínic, University of Barcelona, Barcelona, Spain:** R. Casas, M. Domenech, C. Viñas, S. Castro-Barquero, A.M. Ruiz-León, R. Losno, L. Tarés, A. Jordán, R. Soriano, M. Camafort, C. Sierra, E. Sacanella, A. Sala-Vila, J. M. Cots, I. Sarroca, M. García, N. Bermúdez, A. Pérez, I. Duaso, A. de la Arada, R. Hernández, C. Simón, M.A. de la Poza, I. Gil, M. Vila, C. Iglesias, N. Assens, M. Amatller, LL. Rams, T. Benet, G. Fernández, J. Teruel, A. Azorin, M. Cubells, D. López, J.M. Llovet, M.L. Gómez, P. Climente, L. de Paula, J. Soto, C. Carbonell, C. Llor, X. Abat, A. Cama, M. Fortuny, C. Domingo, A. I. Liberal, T. Martínez, E. Yañez, M. J. Nieto, A. Pérez, E. Lloret, C. Carrazoni, A. M. Belles, C. Olmos, M. Ramentol, M. J. Capell, R. Casas, I. Giner, A. Muñoz, R. Martín, E. Moron, A. Bonillo, G. Sánchez, C. Calbó, J. Pous, M. Massip, Y. García, M.C. Massagué, R. Ibañez, J. Llaona, T. Vidal, N. Vizcay, E. Segura, C. Galindo, M. Moreno, M. Caubet, J. Altirriba, G. Fluxà, P. Toribio, E. Torrent, J. J. Anton, A. Viaplana, G. Vieytes, N. Duch, A. Pereira, M. A. Moreno, A. Pérez, E. Sant, J. Gené, H. Calvillo, F. Pont, M. Puig, M. Casasayas, A. Garrich, E. Senar, A. Martínez, I. Boix, E. Sequeira, V. Aragunde, S. Riera, M. Salgado, M. Fuentes, E. Martín, A. Ubieto, F. Pallarés, C. Sala, A. Abilla, S. Moreno, E. Mayor, T. Colom, A. Gaspar, A. Gómez, L. Palacios, R. Garrigosa.

**Departament of Preventive Medicine and Public Health, University of Granada, Granada, Spain:** L. García Molina, B. Riquelme Gallego, N. Cano Ibañez, A. Maldonado Calvo, A. López Maldonado, E.M. Garrido, A. Baena Dominguez, F. García Jiménez, E. Thomas Carazo, A. Jesús Turnes González, F. González Jiménez, F. Padilla Ruiz, J. Machado Santiago, M.D. Martínez Bellón, A. Pueyos Sánchez, L. Arribas Mir, R. Rodríguez Tapioles, F. Dorador Atienza, L. Baena Camus, C. Osorio Martos, D. Rueda Lozano, M. López Alcázar, F. Ramos Díaz, M. Cruz Rosales Sierra, P. Alguacil Cubero, A. López Rodriguez, F. Guerrero García, J. Tormo Molina, F. Ruiz Rodríguez.

**Bioaraba Health Research Institute, Cardiovascular, Respiratory and Metabolic Area; Osakidetza Basque Health Service, Araba University Hospital; University of the Basque Country UPV/EHU, Vitoria-Gasteiz, Spain:** I. Salaverria, A. Alonso-Gómez, M.C. Belló, L. Tojal, L. Goicolea, C. Sorto, A Goikoetxea, A. Casi Casanellas, M.L. Arnal Otero, J. Ortueta Martínez De Arbulo, J. Vinagre Morgado, J. Romeo Ollora, J. Urraca, M.I. Sarriegui Carrera, F.J. Toribio, E. Magán, A. Rodríguez, S. Castro Madrid, M.T. Gómez Merino, M. Rodríguez Jiménez, M. Gutiérrez Jodra, B. López Alonso, J. Iturralde Iriso, C. Pascual Romero, A. Izquierdo De La Guerra.

**Research Group on Community Nutrition & Oxidative Stress, University of Balearic Islands, Palma de Mallorca, Spain:** M. Abbate, E. Angullo, E. Argelich, M.M. Bibiloni, C. Bouzas, X. Capó, S. Carreres, L. Gallardo, J.M. Gámez, B. García, C. García, A. Julibert, C. Gómez, I. Llompart, A. Martorell, C.M. Mascaró, D. Mateos, M, Monserrat, S. Montemayor, A. Pons, A. Pouso, J. Ramos, V. Ramos, T. Ripoll, T. Rodríguez, L. Sanz, A. Sureda, S. Tejada, L. Ugarriza.

**Virgen de la Victoria Hospital, University of Málaga, Málaga, Spain:** M.R. Bernal López, M. Macías González, J. Ruiz Nava, J.C. Fernández García, A. Muñoz Garach, A. Vilches Pérez, A. González Banderas, A.V. Alarcón-Martín, M. García Ruiz de Mier, J. Alcaide Torres, A. Vargas Candela, M. León Fernández, R. Hernández Robles, S. Santamaría Fernández, J.M. Marín.

**University of Las Palmas de Gran Canaria, Las Palmas, Spain:** J. Álvarez-Pérez, E.M. Díaz Benítez, F. Díaz-Collado, A. Sánchez-Villegas, J. Pérez-Cabrera, L.T. Casañas-Quintana, R.B. García-Guerra, I. Bautista-Castaño, C. Ruano-Rodríguez, F. Sarmiento de la Fe, J.A. García-Pastor, B. Macías-Gutiérrez, I. Falcón-Sanabria, C. Simón-García, A.J. Santana-Santana, J.B. Álvarez-Álvarez, B.V. Díaz-González, J.M. Castillo Anzalas, R.E. Sosa-Also, J. Medina-Ponce.

**Biomedicine Institute (IBIOMED); University of León, and Primary Health Care Management of León (Sacyl), León, Spain: Biomedicine Institute (IBIOMED); University of León, and Primary Health Care Management of León (Sacyl), León, Spain:** S. Abajo Olea, L. Álvarez-Álvarez, M. Rubín García, A. Torres, P. Farias, N. Cubelos, A. Adlbi Sibai, M. Ajenjo, E. Carriedo Ule, M. Escobar Fernández, J.I. Ferradal García, J.P. Fernández Vázquez, C. González Quintana, F. González Rivero, M. Lavinia Popescu, J.I. López Gil, J. López de la Iglesia, A. Marcos Delgado, C. Merino Acevedo, S. Reguero Celada, M. Rodríguez Bul, E. Fernández Mielgo.

**Department of Family Medicine, Distrito Sanitario Atención Primaria Sevilla, Sevilla, Spain:** J.M. Santos-Lozano, L. Miró-Moriano, C. Domínguez-Espinaco, S. Vaquero-Díaz, F.J. García-Corte, A. Santos-Calonge, C. Toro-Cortés, N. Pelegrina-López, V. Urbano-Fernández, M. Ortega-Calvo, J. Lozano-Rodríguez, I. Rivera-Benítez, M. Caballero-Valderrama, P. Iglesias-Bonilla, P. Román-Torres, Y. Corchado-Albalat, L. Mellado-Martín.

**Department of Endocrinology and Nutrition, Hospital Fundación Jimenez Díaz. Instituto de Investigaciones Biomédicas IISFJD. University Autonoma, Madrid, Spain:** A.I. de Cos, S. Gutierrez, S. Artola, A. Galdon, I. Gonzalo.

**Lipids and Vascular Risk Unit, Internal Medicine, University Hospital of Bellvitge- IDIBELL, Hospitalet de Llobregat, Barcelona, Spain:** X. Pintó, A. Galera, M. Gimenez-Gracia, E. de la Cruz, R. Figueras, M. Poch, R. Freixedas, F. Trias, I. Sarasa, M. Fanlo-Maresma, H. Lafuente, M. Liceran, A. Rodriguez-Sanchez, C. Pallarols, E. Gómez-Sanchez, V. Esteve-Luque, J. Monedero, X. Corbella, E. Corbella.

**Department of Endocrinology, IDIBAPS, Hospital Clinic, University of Barcelona, Barcelona, Spain:** A. Altés, I. Vinagre, C. Mestre, J. Viaplana, M. Serra, J. Vera, T. Freitas, E. Ortega, I. Pla, R. Olbeyra.

**Nutritional Control of the Epigenome Group. Precision Nutrition and Obesity Program, Institute IMDEA-Food, CEI UAM+CSIC, Madrid, Spain:** J.M. Ordovás, V. Micó, L. Berninches, L. Díez, M.J. Concejo, J. Muñoz, M. Adrián, Y. de la Fuente, C. Albertos, M.L. Cornejo, C. Cuesta. A. Montero., J. Aroca, B. Cáceres Sánchez, ME. Jiménez Caravera, MA. Aldavero Palacios, S. Conti Fernández, MC. Rodríguez Romero, PJ. Jiménez Pérez, V. Fernández Gutiérrez, Y. De La Fuente Cortes, R. Baños Morras, C. Gómez Almodóvar, P. González Escobar, AM. Ibarra Sánchez, A. Manzanares Briega M. Renata Muñoz Bieber, J. Muñoz Gutiérrez, L. Santos Larregola, C. Cassinello Espinosa, MC. Molins Santos, JM. Rodríguez Buitrago, E. Sánchez Balsalobre, C. Albertos Carrion, C. Cuesta González, L. González Torres, ME. Villahoz Loureiro, E. Arrebola Vivas, AF. Fernández Garcia, ML. Cornejo Alonso, JA. Romo Martin, L. Carabias Jaen, C. Barbero Macías, MA. Blanca De Miguel Oteo, E. Bartolomé Cobeña, MM. Adrián Sanz, MA. Angel Álvaro Sánchez, MD. Cano Pérez, MP. Lopez Morandeira, A. Montero Costa, E. Robles Fernandez, I. Alba Llacer, J. Aroca Palencia, R. Sanz Merino, MJ. Concejo Carranza, A. Garcia Romero, MC. Gómez Tabera, C. Lesmes Lora, J. Zarco Montejo, A. Campo Lopez, ME. Collado Correa, MS. Díaz Moreno, B. Doval Segura, RM. Gómez Quiroga, S. Hernando Gómez, MJ. Martínez Sanz, AM. Yunquera Alonso

**Division of Preventive Medicine, University of Jaén, Jaén, Spain:** J.J. Gaforio, S. Moraleda, N. Liétor, J.I. Peis, T. Ureña, M. Rueda, M.I. Ballesta.

**Department of Endocrinology and Nutrition, Hospital Clínico San Carlos, Instituto de Investigación Sanitaria del Hospital Clínico San Carlos (IdISSC), Madrid, España (Spain para internacionales:** C. Moreno Lopera, C. Aragoneses Isabel, M.A. Sirur Flores, M. Ceballos de Diego, T. Bescos Cáceres, Y. Peña Cereceda, M. Martínez Abad, R. Cabrera Vélez, M. González Cerrajero, M.A. Rubio Herrera, M. Torrego Ellacuría, A. Barabash Bustelo, M. Ortiz Ramos, A. Larrad Sainz.

.
